# Chromatin recruitment of OGG1 requires cohesin and mediator and is essential for efficient 8-oxoG removal

**DOI:** 10.1093/nar/gkaa611

**Published:** 2020-07-25

**Authors:** Emilie Lebraud, Guillaume Pinna, Capucine Siberchicot, Jordane Depagne, Didier Busso, Damiano Fantini, Lamya Irbah, Elena Robeska, Gueorgui Kratassiouk, Jean-Luc Ravanat, Bernd Epe, J Pablo Radicella, Anna Campalans

**Affiliations:** Institut de Biologie François Jacob, Institute of Cellular and Molecular Radiobiology, Université Paris-Saclay, Université de Paris, CEA, 18 route du Panorama, F-92265 Fontenay-aux-Roses, France; Institute for Integrative Biology of the Cell (I2BC), CEA, CNRS, Univ. Paris-Sud, Université Paris-Saclay, F-91198 Gif-sur-Yvette, France; Institut de Biologie François Jacob, Institute of Cellular and Molecular Radiobiology, Université Paris-Saclay, Université de Paris, CEA, 18 route du Panorama, F-92265 Fontenay-aux-Roses, France; Institute of Cellular and Molecular Radiobiology, U1274 INSERM, CEA, 18 route du Panorama, F-92265 Fontenay-aux-Roses, France; Institute of Cellular and Molecular Radiobiology, U1274 INSERM, CEA, 18 route du Panorama, F-92265 Fontenay-aux-Roses, France; Institut de Biologie François Jacob, Institute of Cellular and Molecular Radiobiology, Université Paris-Saclay, Université de Paris, CEA, 18 route du Panorama, F-92265 Fontenay-aux-Roses, France; Institute of Cellular and Molecular Radiobiology, U1274 INSERM, CEA, 18 route du Panorama, F-92265 Fontenay-aux-Roses, France; Institut de Biologie François Jacob, Institute of Cellular and Molecular Radiobiology, Université Paris-Saclay, Université de Paris, CEA, 18 route du Panorama, F-92265 Fontenay-aux-Roses, France; Institute for Integrative Biology of the Cell (I2BC), CEA, CNRS, Univ. Paris-Sud, Université Paris-Saclay, F-91198 Gif-sur-Yvette, France; Univ. Grenoble Alpes, CEA, CNRS IRIG/SyMMES, F-38054 Grenoble, France; Institute of Pharmaceutical and Biomedical Sciences, University of Mainz, Germany; Institut de Biologie François Jacob, Institute of Cellular and Molecular Radiobiology, Université Paris-Saclay, Université de Paris, CEA, 18 route du Panorama, F-92265 Fontenay-aux-Roses, France; Institut de Biologie François Jacob, Institute of Cellular and Molecular Radiobiology, Université Paris-Saclay, Université de Paris, CEA, 18 route du Panorama, F-92265 Fontenay-aux-Roses, France

## Abstract

One of the most abundant DNA lesions induced by oxidative stress is the highly mutagenic 8-oxoguanine (8-oxoG), which is specifically recognized by 8-oxoguanine DNA glycosylase 1 (OGG1) to initiate its repair. How DNA glycosylases find small non-helix-distorting DNA lesions amongst millions of bases packaged in the chromatin-based architecture of the genome remains an open question. Here, we used a high-throughput siRNA screening to identify factors involved in the recognition of 8-oxoG by OGG1. We show that cohesin and mediator subunits are required for re-localization of OGG1 and other base excision repair factors to chromatin upon oxidative stress. The association of OGG1 with euchromatin is necessary for the removal of 8-oxoG. Mediator subunits CDK8 and MED12 bind to chromatin and interact with OGG1 in response to oxidative stress, suggesting they participate in the recruitment of the DNA glycosylase. The oxidative stress-induced association between the cohesin and mediator complexes and OGG1 reveals an unsuspected function of those complexes in the maintenance of genomic stability.

## INTRODUCTION

Cellular DNA is continuously exposed to reactive oxygen species arising from endogenous and exogenous sources. As a consequence, lesions such as modified bases, abasic (AP) sites and single-strand breaks (SSBs) are generated. One of the major base lesions induced by oxidative stress is 8-oxoguanine (8-oxoG), which is recognized and excised by the DNA glycosylase OGG1 that initiates the base excision repair (BER) pathway. Even though 8-oxoG does not induce a significant distortion of the DNA double helix, it has a high mutagenic potential as, during replication, it can favour the incorporation of adenine opposite to it and lead to GC-to-TA transversions. Under basal conditions less than one 8-oxoG is present per million base pairs, whereas its occurrence increases by up to 10-fold upon exposure to oxidative stress (1).

*In vitro* OGG1 scans for 8-oxoG by sliding along naked DNA at a high diffusion rate (2). In cells, however, 8-oxoG detection and removal by OGG1 is the rate-limiting step for BER (1,3), possibly because chromatin limits accessibility to the lesion (4). We have previously shown that oxidative stress-induced 8-oxoG causes the retention of OGG1, together with other BER proteins, in euchromatin regions rich in mRNA and RNA polymerase II, while BER proteins are excluded from heterochromatin (1,5,6). OGG1 residence on chromatin correlates with the repair kinetics of 8-oxoG. An active-site mutant of OGG1 capable of recognizing the lesion but not of excising it, remains tightly associated with chromatin for significantly longer periods. However, OGG1 recruitment to chromatin does not require recognition of the lesion as an OGG1 mutant with no affinity for 8-oxoG is efficiently re-localized to chromatin in response to oxidative stress (1), suggesting that other factors may be involved. Here, we have used a high-throughput siRNA screen to identify proteins involved in the re-localization of OGG1 to chromatin after the induction of 8-oxoG in cellular DNA. Among the candidates, we identified several components of the mediator and cohesin complexes, suggesting a functional link between these two nuclear complexes and BER.

Cohesin and mediator complexes are involved in establishing chromatin organization (7). Initially identified for their role in chromosome cohesion and segregation during mitosis, cohesin also functions in the interphasic nucleus (8), where it regulates the formation and stability of DNA loops (9,10), with different cohesin ring subunit compositions proposed to have different functions (11). Cohesin-binding sites in the genome can be classified into two types: those associated with CCCTC-binding factor (CTCF), and those associated with transcription factors (TFs), mediator and nipped-B-like protein (NIPBL) (7). In humans, mediator is composed of up to 30 subunits that can be divided into four different modules: the head, middle, tail and the CDK8 kinase (CKM) modules. The head and the middle constitute the core of mediator, with the tail and CKM modules playing regulatory roles (12,13). The regions of the genome co-occupied by mediator and cohesin are involved in the formation of loops allowing contacts between promoters and enhancers. Both complexes are particularly enriched at super-enhancers, clusters of enhancers that are densely occupied by master regulator TFs (14,15).

Here, we show that oxidative stress induces a dynamic re-localization of several mediator subunits to euchromatin regions where they colocalize with OGG1. We identify an association between OGG1 and mediator and cohesin complexes, and the requirement of those complexes for the recruitment of OGG1 and other base excision repair proteins to the chromatin fraction. We demonstrate that chromatin retention of OGG1 is required for the efficient excision of the 8-oxoG induced by oxidative stress.

## MATERIALS AND METHODS

### Cell culture

HeLa and HeLa OGG1–GFP cells (1) were cultured in DMEM (Gibco) containing 10% of fetal bovine serum and 1% of penicillin–streptomycin at 37°C with 5% CO_2_. For stable cell line expressing OGG1–GFP, the culture medium was supplemented with 400 μg/ml of G418

### siRNA screen

We performed a systematic, individual and transient gene loss-of-function screening in a HeLa cell line engineered to overexpress hOGG1 fused to GFP, aiming to identify genes regulating oxidative DNA damages recognition by hOGG1. To achieve this, we used an RNA interference library constituted of individual siRNAs (3 siRNAs/genes) arrayed in 384-well format, and designed to specifically target and knock-down 6961 human ‘Druggable’ genes (Human Druggable Genome siRNA Set V4.0, Qiagen). For a detailed description of the siRNA screening see Supplementary Methods and Supplementary Table S1.

### Construction of plasmids

Plasmids used in this study and the sequence of the oligonucleotides used for their construction are indicated in Supplementary Tables S2 and S3 respectively.

The plasmids hOGG1–GFP, XRCC1-GFP and NTH1-GFP have been previously described (5). To obtain the construct OGG1–Dendra2, the sequence encoding hOGG1 was released from the hOGG1–GFP plasmid by restriction with EcoRI + BamHI (in EcoRI buffer). The insert was then ligated into pDendra2-N plasmid (gift from Sébastien Huet) digested by the same enzyme and treated with Fast-AP (ThermoFisher Scientific, Waltham, MA, USA). The ligation reaction was transformed in DH5α-T1R competent cells.

The construct OGG1-HaloTag was generated by replacing the GFP sequence of the OGG1–GFP plasmid by the HaloTag sequence. First, a flexible linker (translation PDPSGAAAAGGSQK) was added between hOGG1 and the GFP in hOGG1–GFP plasmid by PCR. Briefly, hOGG1–GFP plasmid was amplified by PCR with Phusion DNA polymerase (NEB) with SP0054 and SP0055 primers. Upon amplification, the PCR product was treated with DpnI for overnight at 37°C and transformed into DH5α-T1R competent cells. Two additional repeats of the same linker sequence were added by digesting the plasmid with BamHI and treating with T4 DNA polymerase (0.75 U) for single-strand annealing (Li and Elledge, Nat. Methods, 2007) with a hybrid of primer (SP0475 + SP0476) harboring complementary sequences. The resulting plasmid was amplified with SP0674 + SP0675 (see table) and a plasmid harboring Halo tag encoding sequence was amplified with SP0676 + SP0677. After purification on GeneJet PCR purification kit (Thermofisher) to remove all dNTPs, both PCR products were treated with T4 DNA polymerase for single-strand annealing. The annealing reaction was transformed in DH5α-T1R home-made competent cells.

The CDK8-GFP plasmid was constructed by single-strand annealing cloning by combining two PCR products harboring complementary sequences at their ends. Briefly, pEGFP-N1 plasmid (Clontech, Takara Bio Inc. USA, Mountain View, CA) was amplified with SP0625 + SP0626 and HsCdk8-1-464 encoding sequence was amplified from pBabe.puro.CDK8.flag (Addgene plasmid # 19758) with SP0584 + SP0585. After purification on GeneJet PCR purification kit (Thermofisher) to remove all dNTPs, both PCR products were treated with T4 DNA polymerase for single-strand annealing. The annealing reaction was transformed in DH5α-T1R home-made competent cells.

MED12-HaloTag and CDK19-HaloTag were provided by PROMEGA.

### Cell transfections and treatments

Cells were grown on 12 mm coverslides or on μ-slides four or eight wells or the 96-well plates (ibidi Cat# 80427, 80827, 89626) for *microscopy* experiments and on Petri dishes for protein extraction. Cells were transfected with the indicated siRNAs (final concentration 25 nM) using LipoFectamine RNAiMAX (Thermofisher) according to manufacturer's instructions 72 h before treatment. Sequences of the siRNA used are indicated in Supplementary Table S3. When required, plasmids were transfected 24 h before treatment using LipoFectamine 2000 (Thermofisher) according to the manufacturer's instructions. When indicated, cells at ∼80% of confluence were treated with 40 mM potassium bromate (KBrO_3_; Sigma) diluted in DPBS (Sigma) for 45 min at 37°C. For treatments with hydrogen peroxide, cells were incubated in culture medium containing 1 mM H_2_O_2_ (Sigma) and incubated for 4 h at 37°C. After treatment cells were allowed to recover in DMEM for the indicated times before fixation or extraction. For the removal of soluble proteins, cells were washed for 5 min on ice with cold CSK buffer (100 mM NaCl, 300 mM sucrose, 10 mM PIPES pH 6.8, 3 mM MgCl_2_, 0.5% Triton) containing protease inhibitors. The cells were washed twice on ice-cold PBS before fixation in 2% formaldehyde (FA) for 15 min at room temperature. Nuclear DNA was counterstained with 1 μg/ml 4′,6′-diamidino-2-phenylindole (DAPI). The coverslides were mounted in Dako fluorescence mounting medium.

To inhibit the kinase activity of CDK8/CDK19, 100 nM of Cortistatin A (CA) (kindly provided by Henry Efrm Pelish (Harvard University, USA)) was added to the medium two hours before treatment with KBrO_3_ and kept all over the protocol until cell fixation or extraction.

### Protein labeling and immunofluorescence

For visualization of Halotag fused proteins, cells were incubated with 5 μM TMR ligand (Promega) for 15 min. Cells were washed twice with PBS, and further incubated in DMEM for 30 min. Cells were washed twice with PBS before fixation.

For immunofluorescence experiments cells were permeabilized at room temperature in PBS–0.1% Triton for 10 min. Cells were incubated in blocking solution (PBS, 0.1% Triton, 3% BSA, 1% normal goat serum) at 37 °C for 1 h. Cells were subsequently incubated for 1 h at 37°C with the primary antibodies in blocking solution. Antibodies used are indicated in Supplementary Table S4. Cells were then washed three times for 10 min in PBS–0.1% Triton and incubated with secondary antibodies diluted at 1:1000 in blocking solution for 1 h at 37 °C. Nuclear DNA was counterstained with 1 μg/ml DAPI.

For visualization of 8-oxoG, HeLa OGG1–GFP cells grown on coverslips were fixed in acetone:methanol (1:1) and air dried. Cells were hydrated for 15 min in PBS, and DNA was denatured by incubating cells in 2N HCl for 45 min at room temperature. This step was critical in order to allow access of the antibody to the chromatin. Cells were washed three times in PBS and neutralized with 50 mM Tris–HCl pH 8.8 for 5 min before proceeding to the immunofluorescence protocol, as previously described, using the mouse anti-8oxoG (ab48508, abcam) as a primary antibody. Nuclear DNA was counterstained with 1 μg/ml propidium iodide with 50 μg/ml RNAse. For all immunofluorescence experiments, coverslides were mounted in Dako Fluorescent mouting medium.

EdU incorporation was used for labeling of cells in S-phase. Cells were incubated for 30 min with medium containing 5-ethynyl-2′-deoxyuridine (EdU 50 μM) before fixation or CSK pre-extraction (when indicated). EdU was visualized using the Click-iT Plus EdU Alexa Fluor 647 Imaging Kit (Thermo Fisher Scientific, ref. Cat# C10640) following the manufacturer instructions.

### Protein extraction and western blotting

Protein extracts for western blot analysis, cell pellets (∼5 × 10^6^ cells) were resuspended in benzonase buffer (50 mM pH 7.5 Tris–HCl, 20 mM NaCl, 1 mM MgCl_2_, 0.1% SDS, benzonase 0.01 U/ml) containing protease inhibitors, sonicated in a Bioruptor^®^ bath (pulses 30′ on/30′ off for 10 min at maximum intensity) and centrifuged at 12 000 rpm for 5 min at 4°C. When removal of soluble proteins was required, cell pellets (∼5 × 10^6^ cells) were incubated for 5 min at 4°C in 1 ml ice-cold CSK buffer (100 mM NaCl, 300 mM sucrose, 10 mM PIPES pH 6.8, 3 mM MgCl_2_) containing 0.5% triton X-100 and protease inhibitors. After centrifugation at 12 000 rpm for 5 min at 4°C, pellets were washed twice with 1 ml ice-cold CSK. The resulting pellets were resuspended in Laemmli buffer and boiled. Between 20 and 40 μg of proteins were loaded onto 4–15% Mini-PROTEAN TGX Precast Protein Gels (Biorad). Proteins were separated by electrophoresis at 30 mA and transferred to Trans-Blot membranes (Biorad) for 10 min at 1.3A using a Trans-Blot® Turbo machine (Biorad). Before addition of primary antibodies, membranes were blocked overnight in PBS containing 0.1% Tween 20 (PBST) and 3% milk. The next day, the membrane was washed three times with 1× PBST and incubated with the primary antibody for 1 h at room temperature. Antibodies used are indicated in Supplementary Table S4. After three washes with 1× PBST, the membrane was incubated with the secondary antibodies for 1 h at room temperature. Western blots were revealed with the ODYSSEY CLx.

### Co-immunoprecipitation

Cell pellets (∼20 × 10^6^ cells) were extracted in lysis buffer (20 mM pH 7.5 Tris–HCl, 150 mM NaCl, 1 mM EDTA, 0.1% NP40, 1 Mm DTT, 1 mM MgCl_2_, benzonase 0.01 U/ml) containing protease inhibitors, sonicated in a Bioruptor^®^ bath (pulses 30′ on/30′ off for 2 min at maximum intensity) and centrifuged at 12 000 rpm for 5 min at 4°C. One mg of cleared cell lysate was incubated with 2 μg of a specific antibody (targeted protein or control IgG) for 30 min on ice without agitation. Antibodies used are indicated in Supplementary Table S4. Then, 100 μl of Dynabeads (11204D, Invitrogen) were added to the samples and the mix was incubated for 1 h at 4°C on an orbital rotor. After three washing steps with 0.1% NP40 buffer, beads were denatured in 25 μl 1× Laemmli buffer for 5 min at 95°C. Finally, immunoprecipitations and 50 μg of input were loaded and separated on 4–15% SDS-PAGE gel and proteins were revealed with the same conditions than for western blotting.

### 8-oxoG DNA glycosylase assay

Protein extracts were prepared by resuspending cell pellets in a buffer containing 20 mM Tris–HCl, 250 mM NaCl and 1 mM EDTA, sonicated in a Bioruptor^®^ bath (pulses 30′ on/30′ off for 10 min at maximum intensity) and centrifuged at 20 000 × g for 30 min at 4°C. A 34-mer oligonucleotide containing an 8-oxoG at position 16 and labeled at the 5′ end with Cy5 was hybridized to its complementary oligonucleotide containing a cytosine opposite of the 8-oxoG:C-labeled duplex. In a standard reaction, mixture protein extracts (4 μl final volume) were added to a 10 μl reaction mixture containing 150 fmol of the 8- oxoG:C labeled duplex in 20 mM Tris–HCl, 1 mM EDTA, 200 mM NaCl, 1 mg/ml BSA and 5% glycerol. After 1 h at 37°C, NaOH (0.1N final concentration) was added, and the mixture was further incubated for 15 min at 37°C and stopped by adding 4 μl of formamide dye and heating for 5 min at 95°C. The products were resolved by denaturing 7 M urea–20% PAGE. Gels were scanned with a Typhoon imager (Amersham) and band intensities were quantified with ImageQuant^®^ software. The control reaction containing the oligonucleotide incubated without protein extract was used to calculate the background. The percentage of 8-oxoG cleaved is calculated, after subtraction of background, as follows: % cleavage = product/(product + substrate) × 100.

### Confocal microscopy

Image acquisitions of fixed cells were performed with a Nikon A1 confocal microscope, using ×60 oil immersion 1.3 NA or ×20 0.8 NA objectives. For quantification of a high number of cells, mosaics of 3 × 3 or 4 × 4 with 10% stitching were performed.

Photoconversion experiments in living cells were performed with a Nikon A1 inverted confocal microscope equipped with an environmental chamber allowing the control of temperature, humidity and gas mixture. Photoconversion was performed in a 1 μm^2^ squared region localized outside nucleoli by irradiation with a 405 nm laser for 2 s. For acquisition of Dendra2 green and red fluorescence, resonant laser at 488 and 561 nm were used. Before and after photoconversion, confocal image series of one mid z-section were acquired at a frame rate of 2 images/s for a period of 30 s. Confocal image series were typically recorded with a frame size of 512_512 pixels using a PLAN APO 60×/1.4 oil objective. For evaluation of OGG1 protein dynamics, red fluorescence intensities of the photoconverted region were measured and normalized to the immediately post-irradiation value. Experiments were repeated at least three times and an average of 10 cells from a representative experiment are displayed in the corresponding graphs.

FLIM-FRET was performed using a Leica SP8 confocal microscope with a 60× oil immersion 1.3 NA objective. GFP/mCherry or GFP/Halotag fluorophores were used as FRET pairs. HeLa cells were grown on four wells μ-slides 4 (*ibidi*) and transfected with CDK8-GFP and OGG1-Halotag (or OGG1-mCherry) plasmids as previously described. Experiments were performed 48 h after transfection. When indicated, cells at ∼80% of confluence were treated with 40 mM potassium bromate (KBrO_3_; Sigma) diluted in DPBS (Sigma) for 45 min at 37°C. Cells were allowed to recover for 3 h before to be fixed in 2% FA ± CSK buffer. Fluorescence lifetimes were calculated for a particular region of interest (ROI), representing the nucleus, using Symphotime software. For each condition, around 10 cells per condition were analyzed.

### Image analysis, quantifications and statistics

Image adjustement, montages and Plot profiles were obtained with ImageJ. Correlation coefficients and measurements of fluorescence intensity analysis were done with ImageJ and NIS-Elements software. The number of cells analysed is mentioned in the Figure legends. For the measurements of fluorescence intensity, DAPI or PI staining were used to generate a mask of the nucleus that was applied to the other channels. Fluorescence Intensity values measured in the control cells were set to 1 and used for normalization. Statistical analysis performed are indicated in the legend to the figures and were done using Prism software (GraphPad Software). Significance is denoted as (***) for *P* < 0.001, and (****) for *P* < 0.0001.

Details of the reagents and software used in this study are indicated in Supplementary Tables S5 and S6 respectively.

## RESULTS

### Cohesin and mediator are essential for OGG1’s association with chromatin

**Figure 1.**
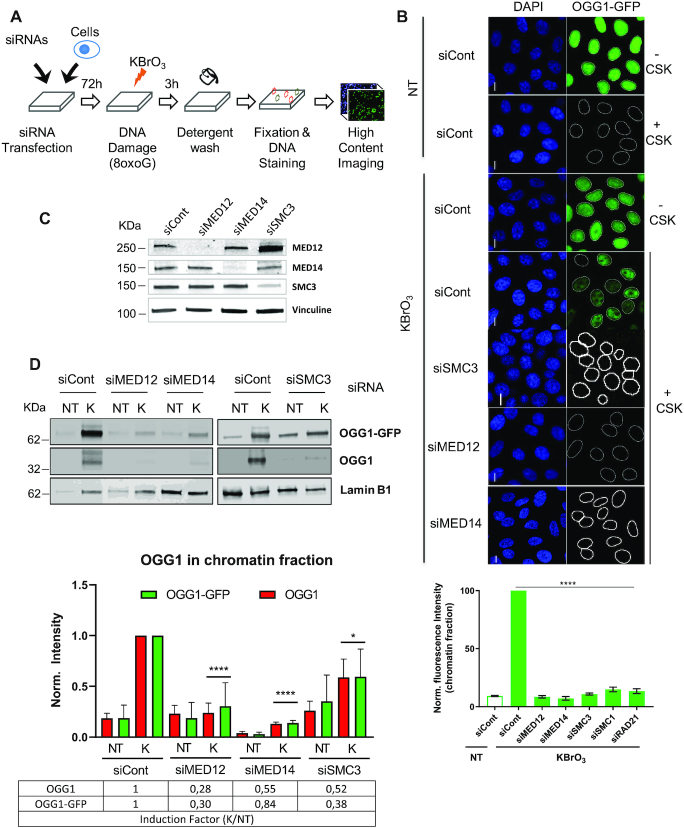
Cohesin and mediator are essential for OGG1’s association with chromatin. (**A**) Cell-based assay setup for siRNA high-throughput screening (details in the supporting material). (**B**) Upper panel, HeLa cells stably expressing OGG1–GFP were transfected with the indicated siRNAs and the presence of OGG1–GFP in KBrO_3_^–^ or non-treated (NT) cells was evaluated before or after removal of the soluble fraction by CSK pre-extraction. Nuclei were stained with DAPI. Scale bar, 5 μm. Lower panel, quantification of the OGG1–GFP on chromatin. At least 1000 cells were analysed after CSK extraction for each siRNA and the experiment was repeated three times. Values obtained in cells transfected with the siRNA control were set to 1 and used for normalization. Error bars represent SEM. Results are expressed as the normalized mean GFP fluorescence ± SEM. Statistical analysis with a Kruskal–Wallis test. (****) *P* < 0.0001. (**C**) Protein extracts prepared from cells transfected with siRNAs against MED12, MED14 or SMC3 were analysed by western blot to check for the efficiency of the siRNAs. (**D**) Effect of depletion of MED12, MED14 and SMC3 on the presence of OGG1–GFP and OGG1 in the chromatin fractions of untreated cells (NT) and cells exposed to KBrO_3_ (K). Cells were washed with CSK buffer to remove soluble proteins and the levels of chromatin associated OGG1 were evaluated by western blot. Lamin B1 was used as a loading control. The graph represents quantification of at least three independent experiments. Intensity was normalized to the levels measured in the control cells exposed to KBrO_3_. KBrO_3_ enrichment of the indicated proteins is represented as a ratio of the levels measured in KBrO_3_ compared to those in NT cells. Ratios are normalieed to the values obtained in the cells transfected with the siControl set to 1. Error bars represent SD. Statistical analysis was performed with an unpaired *t*-test for both OGG1 and OGG1–GFP comparing the effect of each siRNA to the siRNA Control. (****) *P* < 0.0001; (*) *P* <0.05.

We have previously shown that an oxidative stress inducing large amounts of 8-oxoG in genomic DNA results in the re-localization of OGG1 to chromatin (1). In order to identify proteins involved in the association of OGG1 with chromatin, we performed a siRNA-based loss-of-function screening in a HeLa cell line expressing hOGG1 fused to GFP. Briefly, cells were transfected with individual siRNAs from a library targeting 6921 human genes (3 siRNAs/gene), then exposed to KBrO_3_ to induce 8-oxoG in the cellular DNA. Three hours after exposure to KBrO_3_, the time point at which accumulation of OGG1 to chromatin reaches its maximum levels (1), soluble proteins were removed by pre-extraction with cytoskeleton buffer (CSK) and cells were fixed and stained with Hoechst 33342. The chromatin bound fraction of OGG1–GFP, reflecting an equilibrium between recruitment, retention and release, was quantified by high-content imaging (Figure 1A). Chromatin levels of OGG1 in response to gene knock-down were evaluated by the integrated GFP-specific intensity remaining in the nucleus (Nuclear-Specific Fluorescence, *NSF*) and by the percentage of GFP positive cells (%GFP). The primary screen identified 604 candidate genes affecting the enrichment of OGG1 in the chromatin fraction upon KBrO_3_ treatment (see details in Supplementary data (16,17). Candidate genes were retested in a secondary screen that assayed siRNAs that affected the re-localization of OGG1 and not its expression level, using four individual siRNAs/gene, and 168 genes were re-confirmed as hits. Of those, depletion of 81 resulted in reduced association of OGG1 with chromatin, while the opposite effect was observed for the rest (Supplementary Table S1). Among the first group were those targeting three components of the cohesin ring, SMC3, SMC1 and RAD21, and the MED14 or MED12 mediator subunits (Figure 1B, Supplementary Figure S1A and B). Interestingly, both complexes have not only been implicated in chromatin organization but there is also evidence suggesting they do so by functionally interacting (7). We therefore decided to further investigate their potential role in facilitating the recognition and repair of oxidized bases by the DNA glycosylase in the nuclear context. We confirmed that the siRNAs efficiently reduced the levels of the corresponding proteins (Figure 1C, Supplementary Figure S1B) and resulted in substantial impairment of OGG1–GFP association with chromatin after oxidative stress as determined by immunofluorescence (Figure 1B, Supplementary Figure S1C). Subcellular fractionation and immunoblots further confirmed the requirement for mediator and cohesin complexes for the association of OGG1–GFP with chromatin and showed that the untagged OGG1 displayed the same dependence for its re-localization to chromatin (Figure 1D). In order to evaluate if deficiency on these complexes induced a complete lack of or just a delay in the accumulation of OGG1 on chromatin, we evaluated OGG1 chromatin levels at later times. As shown in Supplementary Figure S1C, in cells depleted for mediator or cohesin subunits, OGG1 levels on chromatin remained very low 6 and 9 h after the KBrO_3_ treatment, while in control cells OGG1 is still detectable in significant amounts at 6 h and released from chromatin at 9 h. We then asked if the mediator- and cohesin-dependent accumulation of OGG1 on chromatin is a general response to oxidative stress. To address this question, we treated cells with H_2_O_2_. In that case, we also observed an association of the DNA glycosylase with chromatin that was dependent on the presence of MED12, MED14 and SMC3 subunits (Supplementary Figure S1D).

Given that most cohesin functions are cell-cycle-dependent, we tested whether the requirement for cohesin and mediator in OGG1 re-localization to chromatin depended on the cell-cycle phase. For that, we monitored OGG1 presence on chromatin after KBrO_3_ exposure in parallel to cell-cycle specific markers. We observed a clear recruitment of OGG1–GFP to chromatin in G1, S, and G2 (Supplementary Figure S2A and B), indicating that OGG1 recruitment in cells exposed to KBrO_3_ was not dependent on the cell-cycle phase. In cells transfected with siRNAs against subunits of mediator (MED12, MED14) or cohesin (SMC3), the recruitment of OGG1 was substantially decreased in all three phases of the cell cycle (Supplementary Figure S2C). These results established that recruitment of OGG1 to chromatin is dependent on both cohesin and mediator throughout the cell cycle.

### OGG1 immobilization on euchromatin requires cohesin and mediator

**Figure 2.**
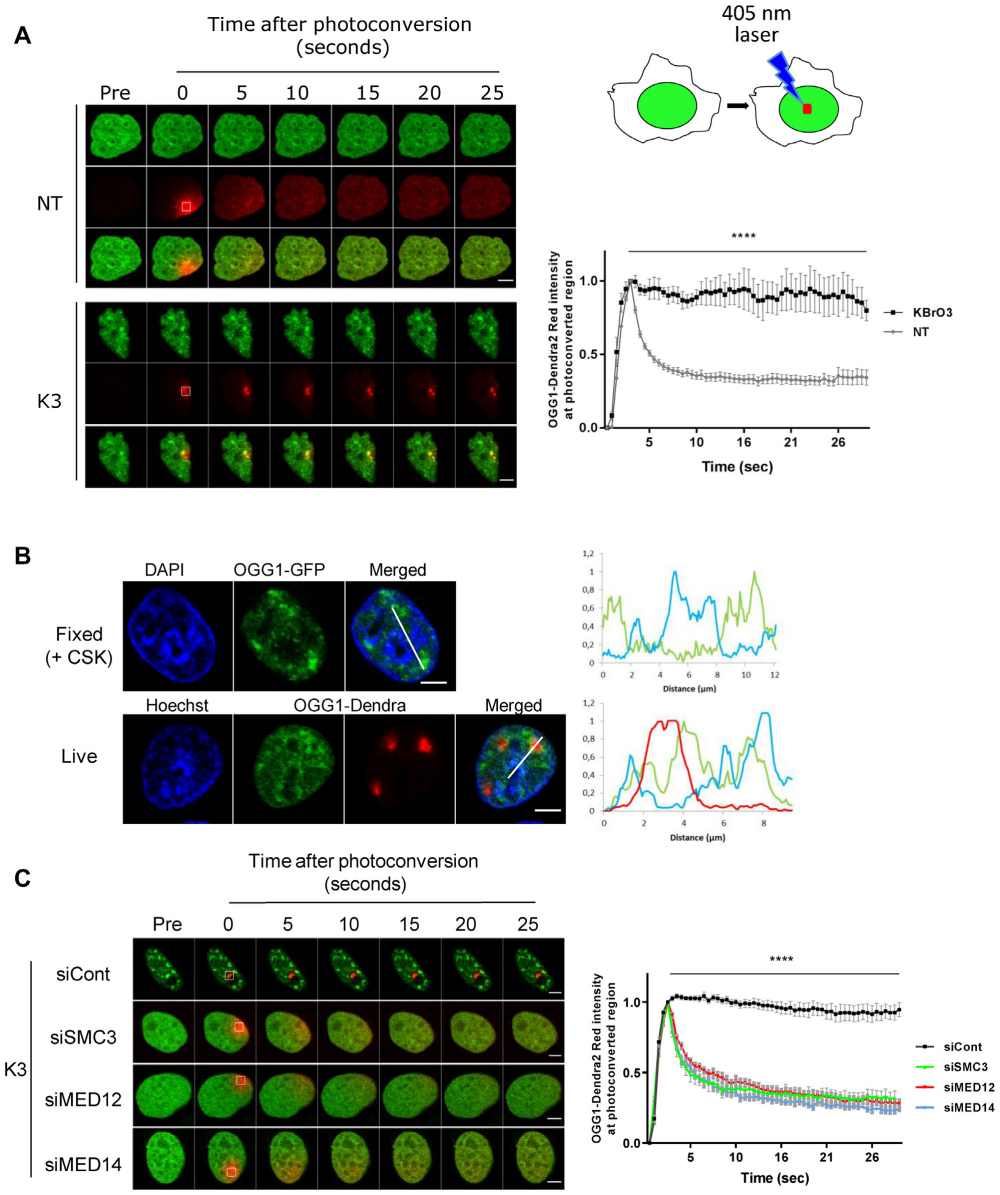
OGG1 immobilization on euchromatin requires cohesin and mediator. (**A**) OGG1–Dendra2 was photoconverted from green to red using the 405 nm laser and both green and red signals were followed over time. Images obtained before photoconversion or at different times after photoconversion are shown. Red signal was monitored in the photoconverted region in both non-treated cells (NT) and 3 h after exposure to KBrO_3_ (K3). Graph shows quantification of the red fluorescence in 10 cells from two independent experiments. Statistical analysis was performed using multiple *t*-test (****) *P* < 0.0001. (**B**) HeLa cells expressing OGG1–GFP or OGG1–Dendra2 were exposed to KBrO_3_ and protein localization evaluated after CSK pre-extraction or photoconversion respectively. DNA (blue) is labeled with DAPI in fixed cells or Hoechst 33345 in living cells and used as an indicator of chromatin compaction. For the OGG1–Dendra2 experiment Hoechst 33345 was added after photoconversion. Plot profiles show the retention of OGG1 in regions showing a weak DAPI/Hoechst staining corresponding to euchromatin domains. (**C**) Protein dynamics of photoconvertible OGG1–Dendra2 in cells depleted for SMC3, MED12 or MED14 3 h after KBrO_3_ treatment (K3). Graph shows quantification of red fluorescence in 10 cells for each siRNA from a representative experiment. The experiment was repeated three times. Statistical analysis was performed using multiple *t*-test (****) *P* < 0.0001. Scale bars: 5 μm.

Our previous results suggest a change in the dynamics of OGG1 induced by oxidative stress (Figure 1, (1,5)). To follow the behavior of OGG1 in living cells, the protein was fused to the green-to-red photo-convertible fluorescent protein Dendra2. Illumination of a 1 μm^2^ nuclear region with a 405 nm laser induced the photo-conversion of the irradiated fraction of OGG1–Dendra2 molecules allowing to follow the sub-nuclear dynamics of the protein in real time. In non-treated cells, photo-converted OGG1–Dendra2 diffused rapidly throughout the nucleus, indicating that most OGG1 molecules move freely within the nucleoplasm. In contrast, three hours after exposure to KBrO_3_, a photo-converted spot of OGG1–Dendra2 remained stable, suggesting that OGG1 was immobilized on chromatin upon oxidative stress (Figure 2A), a result consistent with our observation that the protein becomes resistant to pre-extraction with CSK buffer. When cells were allowed to recover from oxidative stress for 22 hours, photoconverted OGG1–Dendra2 had recovered mobility to the level observed in non-damaged cells (Supplementary Figure S3). Immobilized OGG1–Dendra2 in cells treated with KBrO_3_ was limited to regions with weaker Hoechst staining, corresponding to euchromatin (Figure 2B), consistent with the observed recruitment of OGG1 to those regions (1). In cells depleted of cohesin subunit SMC3 or mediator subunits MED14 or MED12, photoconverted OGG1–Dendra2 diffused freely in the nucleus even after KBrO_3_ treatment (Figure 2C). Thus, cohesin and mediator are essential for the stable association of OGG1 with euchromatin regions in response to oxidative stress.

### 8-oxoG excision is impaired in the absence of mediator and cohesin subunits

We have previously shown a good correlation between the presence of OGG1 in the chromatin fraction and the kinetics of removal of 8-oxoG (1), suggesting that OGG1 retention on chromatin is linked to the 8-oxoG excision process. Since mediator and cohesin are required for OGG1 presence on chromatin after an oxidative stress, cells depleted for those complexes’ subunits should be affected in their capacity to repair 8-oxoG. To test this hypothesis, we set up a protocol to detect 8-oxoG levels by immunofluorescence using an antibody against 8-oxoG. In order to validate this approach, the kinetics of repair of KBrO_3_-induced 8-oxoG in OGG1–GFP expressing cells was followed in parallel by immunofluorescence, HPLC/MS–MS (18) and alkaline elution (19). As shown in Supplementary Figure S4A, all three techniques yielded very similar kinetics of 8-oxoG removal that were consistent with previous reports (1). Moreover, both alkaline elution (3) and HPLC/MS–MS (Supplementary Figure S4B) experiments showed that overexpression of OGG1 results in an accelerated removal of 8-oxoG. The simultaneous detection of 8-oxoG and GFP in immunofluorescence experiments showed that this approach was sensitive enough to observe a significantly lower 8-oxoG signal three hours after exposure to KBrO_3_ in cells expressing higher levels of OGG1–GFP (Supplementary Figure S4C), in agreement with the faster repair kinetics observed in those cells by HPLC/MS–MS (Supplementary Figure S4B). Taken together these results support the use of the immunofluorescence detection of 8-oxoG as a mean to determine the *in vivo* repair kinetics of this lesion.

**Figure 3.**
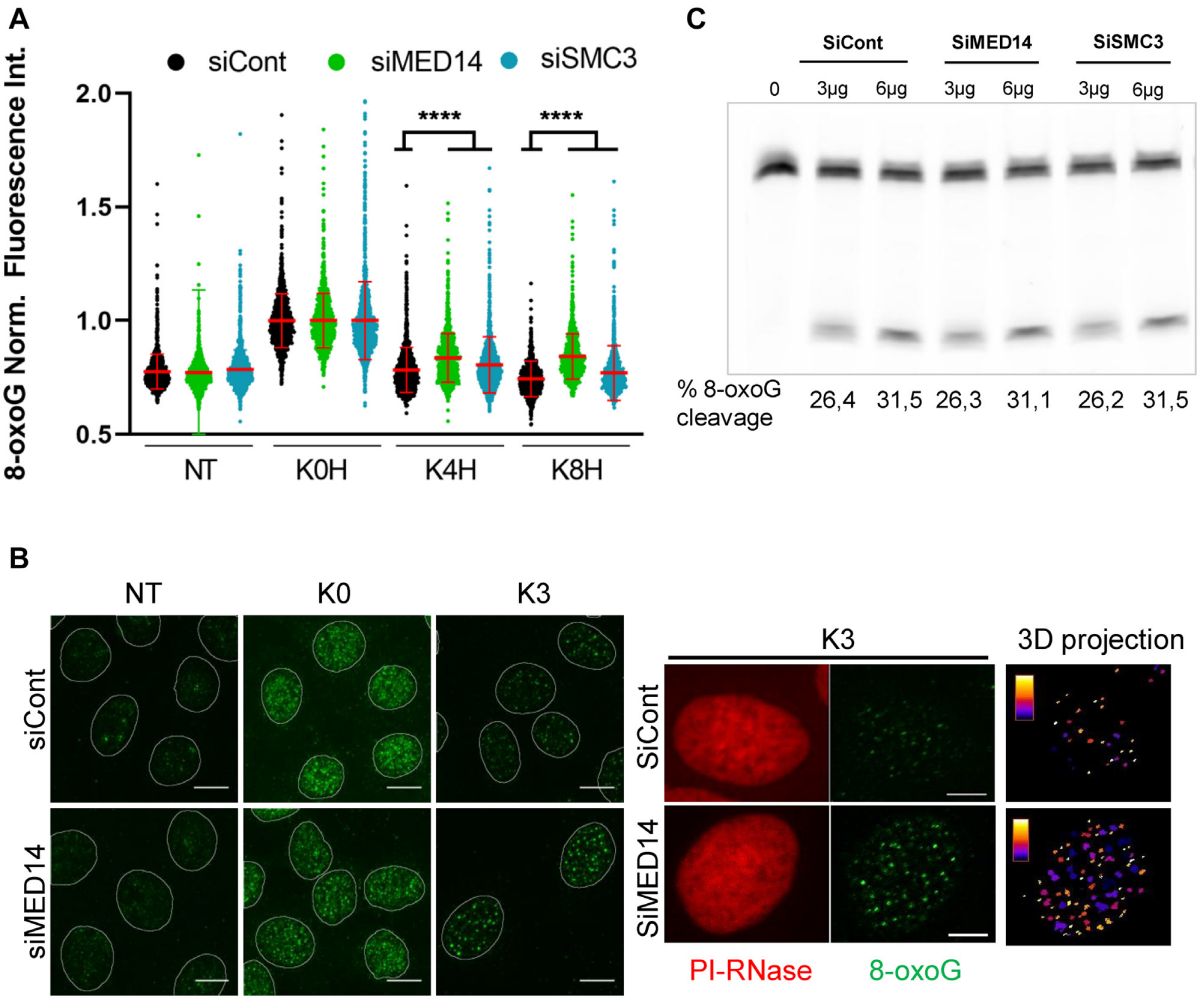
8-oxoG excision is impaired in the absence of mediator or cohesin. (**A**) Relative 8-oxoG levels were determined by immunofluorescence using an antibody against the lesion before and at different times after exposure to KBrO_3_ in control cells or cells depleted for SMC3 or MED14. NT = non-treated, K0H, K4H and K8H = immediately, 4 and 8 h after treatment, respectively. More than 1000 cells were analysed for each of the conditions and presented results correspond to a representative experiment out of three independent ones. The mean of 8-oxoG measured immediately after exposure to KBrO_3_ (K0H) for each siRNA was set to 1 and used for normalization. Statistical analysis used a Kruskal–Wallis test. (****) *P* < 0.0001. (**B**) Representative images of 8-oxoG (green) detected by immunofluorescence in non-treated (NT) and KBrO_3_ treated cells, at 0 (K0) or 3 (K3) hours after the treatment in control cells or cells depleted in MED14. A maximal projection of a z-stack (49 slices every 150 nm) is shown. The mask of the nucleus obtained using propidium iodide (PI) staining is shown. Higher magnifications for PI and 8-oxoG are shown in the right panels. A 3D projection of the 8oxoG foci through the entire nuclear volume is shown. The color scale illustrates the volume of the foci from smaller (white) to bigger (blue). Scale bar: 10 μm. (**C**) OGG1 activity in total cell extracts from cells depleted for MED14 or SMC3 compared to control cells. Cleavage of an 8oxoG-containing oligonucleotide by increasing amount of protein total extracts.

We thus monitored the presence of unrepaired 8-oxoG in non-treated cells and at different times after exposure to KBrO_3_ in both control cells and in cells depleted for either MED14 or SMC3, in which OGG1 is not enriched on chromatin. While similar levels of 8-oxoG were observed immediately after the treatment (K0) in control cells and in cells depleted for MED14 or SMC3, significantly higher levels of 8-oxoG were detected in the cells deficient for either complex at 4 and even 8 hours after exposure to KBrO_3_ (Figure 3A, B and Supplementary Figure S4D). To exclude the possibility that the defect in the removal of 8-oxoG was due to reduced OGG1 DNA glycosylase activity, protein extracts from cells transfected with siControl, siMED14 or siSMC3 were incubated with an oligonucleotide harboring an 8-oxoG residue. Equivalent 8-oxoG excision activities were measured in all three cell extracts (Figure 3C), thus indicating that depletion of these subunits did not affect the enzymatic activity of OGG1 *per se*. Taken together, these results indicate that mediator and cohesin complexes, and thus the association of OGG1 with chromatin in response to oxidative stress, are required for the efficient removal of the 8-oxoG.

### Co-recruitment of mediator CKM subunits and OGG1

**Figure 4.**
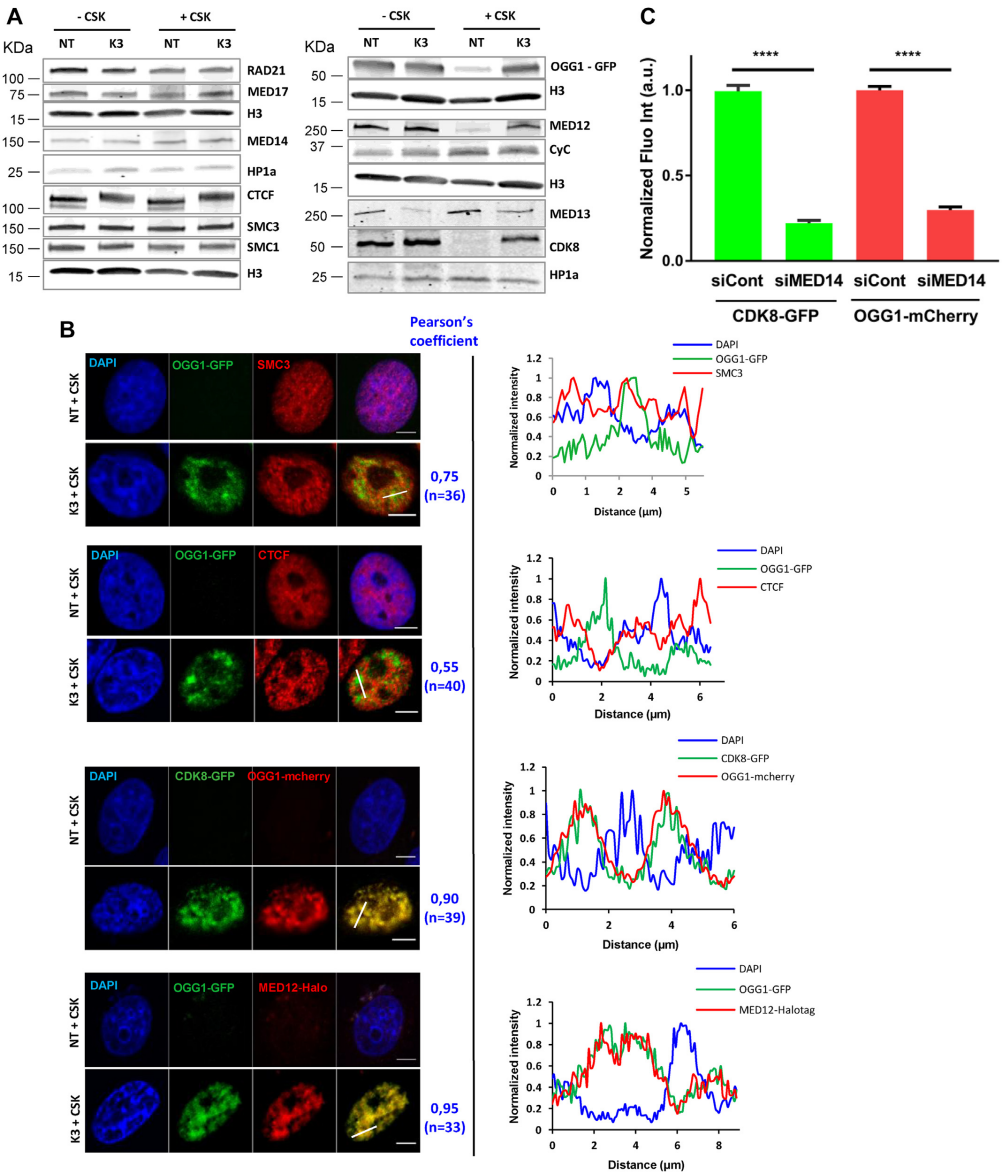
Co-recruitment of mediator CKM subunits and OGG1 to the same nuclear regions. (**A**) Subcellular fractionation of non-treated (NT) and KBrO_3_ (K3)-treated OGG1–GFP cells. Whole cell extract (–CSK) or the insoluble fractions (+CSK) were analysed by western blot using the indicated antibodies. HP1α or H3 were used as loading controls. (**B**) Distribution patterns of OGG1–GFP, SMC3, CTCF, CDK8-GFP and MED12-Halotag in non- (NT) and KBrO_3_- (K3) treated cells. Prior to fixation, soluble proteins were removed with CSK. Scale bar: 5 μm. Pearson correlation coefficient was calculated for the indicated number of cells from at least two independent experiments. Plot profiles along the lines in the merged image are shown. (**C**) Quantification of OGG1-mCherry and CDK8-GFP fluorescence intensities after KBrO_3_ treatment and CSK washing in control cells or cells depleted for MED14. More than 4000 cells were analysed from three independent experiments. Error bars indicate SEM. Statistical analysis involved a Kruskal–Wallis test. (****) *P* < 0.0001.

Since mediator and cohesin are required for OGG1 association with chromatin after oxidative stress, we tested whether the localization of these complexes was affected by KBrO_3_ treatment. Subcellular fractionation and Western blot analysis revealed that all three cohesin ring subunits and the mediator core subunits MED14 and MED17 were constitutively present in the CSK-resistant fraction and remained so after induction of the oxidative stress (Figure 4A).

We next assessed whether the localization of the CKM module of mediator was affected by oxidative stress. CKM is composed of the kinase CDK8, Cyclin C, MED12 and MED13, and is only transiently associated with the mediator core (12,13). The MED13 and cyclin C subunits were present in the chromatin fraction both before and after oxidative stress, similarly to MED14 and MED17 from the core domain. In contrast, CDK8 and MED12 were highly enriched in the chromatin fraction as a response to oxidative stress (Figure 4A), mirroring the behavior of OGG1. No difference in the protein levels was observed in whole cell extracts between non-treated and KBrO_3_-treated cells, indicating that the increase in their levels detected in the chromatin fraction in cells exposed to oxidative stress was not due to an increase in expression or stability, but rather to a difference in subcellular localization. Thus, CKM subunits show markedly different behaviors with respect to their response to oxidative stress, with two of the subunits, CDK8 and MED12, being recruited from the nucleoplasm to the chromatin fraction.

Immunofluorescence experiments confirmed the presence of SMC3 (Figure 4B) and the mediator core subunit MED17 (Supplementary Figure S5A) in the chromatin fraction independently of oxidative stress. Analysis of the fluorescence profiles of OGG1 and SMC3 in the insoluble fraction of cells exposed to KBrO_3_ showed a partial co-localization of the two proteins (Figure 4B). Cohesin is known to interact with mediator and CTCF in a mutually exclusive manner (7), suggesting that it has distinct functions depending on the proteins it associates with. Immunoblot analysis showed that CTCF and the cohesin subunits SMC1 and SMC3 were present in the CSK-resistant fraction at the same levels before and after treatment of the cells with KBrO_3_ (Figure 4A). Comparison by confocal microscopy of OGG1 and CTCF fluorescence profiles in the chromatin fraction of KBrO_3_-treated cells revealed very limited overlap between the proteins (Pearson's correlation coefficient of 0.5) (Figure 4B). In contrast, mediator subunits MED12 and CDK8 showed substantial co-localization with OGG1 after exposure to KBrO_3_ (Pearson's correlation coefficient of 0.9). Co-staining of DNA using DAPI revealed that MED12 and CDK8 co-localize with OGG1 in the less dense DAPI stained regions corresponding to euchromatin (Figure 4B).

The CKM subunits have been proposed to have roles that are independent of the rest of mediator (20). To determine if the recruitment of the CKM subunits MED12 and CDK8 to chromatin upon exposure of cells to KBrO_3_ was dependent on the mediator core, we knocked down MED14, the subunit essential for maintaining the core domain architecture (21), and looked for the presence of MED12 and CDK8 in the chromatin fraction after oxidative stress. As for OGG1, we observed that enrichment of CDK8 and MED12 in the chromatin fraction in KBrO_3_-treated cells was impaired by depletion of MED14 (Figure 4C, Supplementary Figure S5B). Thus, while the mediator core subunits together with CKM subunits MED13 and Cyclin C were constitutively present in the chromatin fraction, MED12 and CDK8 were retained on chromatin, where they co-localized with OGG1, only after oxidative stress. The retention of CDK8, MED12 and OGG1 requires the mediator core, indicating that this process is not an independent role of the CKM subunits.

### CDK8 presence but not its enzymatic activity is required for OGG1 association with chromatin

**Figure 5.**
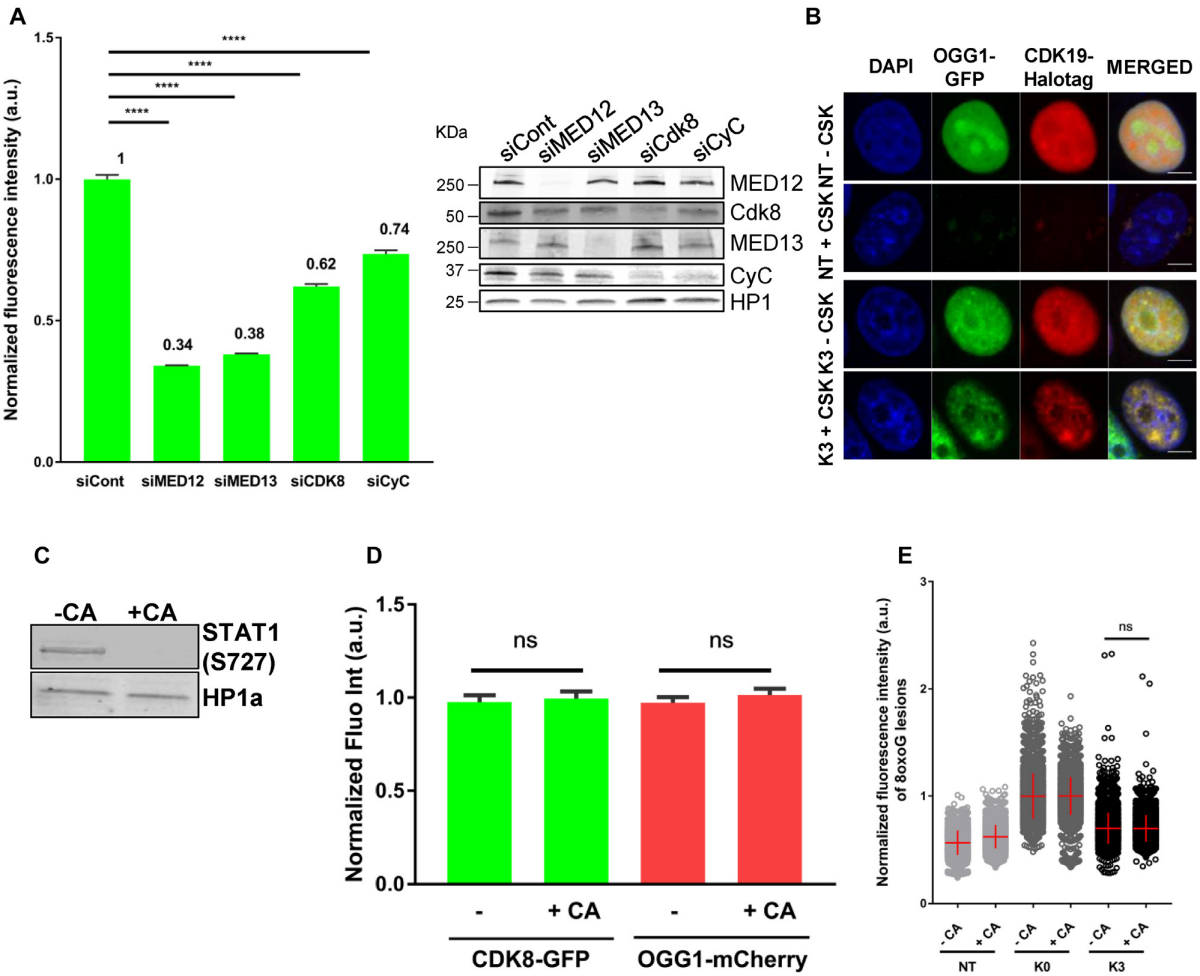
CDK8 presence but not its enzymatic activity is required for recruitment of OGG1. (**A**) Quantification of OGG1–GFP fluorescence intensity in non- (NT) and KBrO_3_- (K3) treated after CSK washing in HeLa OGG1–GFP cells transfected with a siRNA control or siRNAs against the indicated mediator subunits. More than 2000 cells were analysed from two independent experiments. Error bars indicate SEM. Statistical analysis involved a Kruskal–Wallis test. (****) *P* < 0.0001. Specificity and efficiency of the siRNAs was validated by Western blot. (**B**) Distribution patterns of OGG1–GFP/CDK19-Halotag in non- (NT) and KBrO_3_- (K3)-treated cells. Prior to fixation, soluble proteins were removed with CSK when indicated. Scale bar: 5 μm. (**C**) Efficiency of the CDK8–CDK19 inhibitor CA was evaluated by the phosphorylated state of STAT1 (S727). (**D**) Quantification of OGG1-mCherry and CDK8-GFP fluorescence intensities in KBrO_3_ treated cells after CSK washing, in HeLa cells incubated or not with a CDK8 inhibitor (CA). 2900 cells from three independent experiments were analysed. Error bars indicate SEM. Statistical analysis involved a Kruskal–Wallis test. (**E**) Quantification of 8-oxoG fluorescence intensity in non- (NT) or KBrO_3_ treated HeLa OGG1–GFP cells fixed just after treatment (K0) or after 3 h of recovery (K3), in the presence or absence of the CDK8/CDK19 inhibitor CA. More than 4000 cells were analysed from three different experiments. The mean of 8-oxoG measured immediately after exposure to KBrO_3_ (K0H) for each condition was set to 1 and used for normalization. Statistical analysis involved a Kruskal–Wallis test.

To explore the role of the CKM subunits in the recruitment of OGG1 to chromatin after damage induction, individual siRNAs were used to deplete cells of MED12, MED13, CDK8 or Cyclin C. All showed reduced retention of OGG1 in chromatin (Figure 5A). CDK8 and its paralogue CDK19, which was also recruited to chromatin and co-localized with OGG1 in response to oxidative stress (Figure 5B), are the only mediator subunits having enzymatic activity (22). To test whether their kinase activities influenced the nuclear dynamics of OGG1 or CDK8, we exposed HeLa cells co-transfected with OGG1-mCherry and CDK8-GFP to cortistatin A (CA), a highly selective CDK8/CDK19 kinase inhibitor (23,24) (Figure 5C), and monitored the association of both proteins with chromatin. Regardless of the presence of the inhibitor, treatment with KBrO_3_ resulted in the enrichment of OGG1 and CDK8 in the chromatin fraction (Figure 5D) indicating that the CDK8-CDK19 kinase activities were not required for recruitment. The CDK8–CDK19 kinase activities could still be necessary for the excision of 8-oxoG. To test this possibility, we examined the effect of CA on 8-oxoG removal. We found no significant differences in the repair of KBrO_3_-induced 8-oxoG between control and CA-treated cells (Figure 5E), indicating that the CDK8/19 kinase activity is not required for the excision of 8-oxoG. Collectively, our results reveal that all CKM subunits are necessary for OGG1 recruitment to chromatin and in the case of CDK8 it is the physical presence of the subunit, but not its enzymatic activity that is required.

### Oxidative stress induces the association of OGG1 with mediator and cohesin

**Figure 6.**
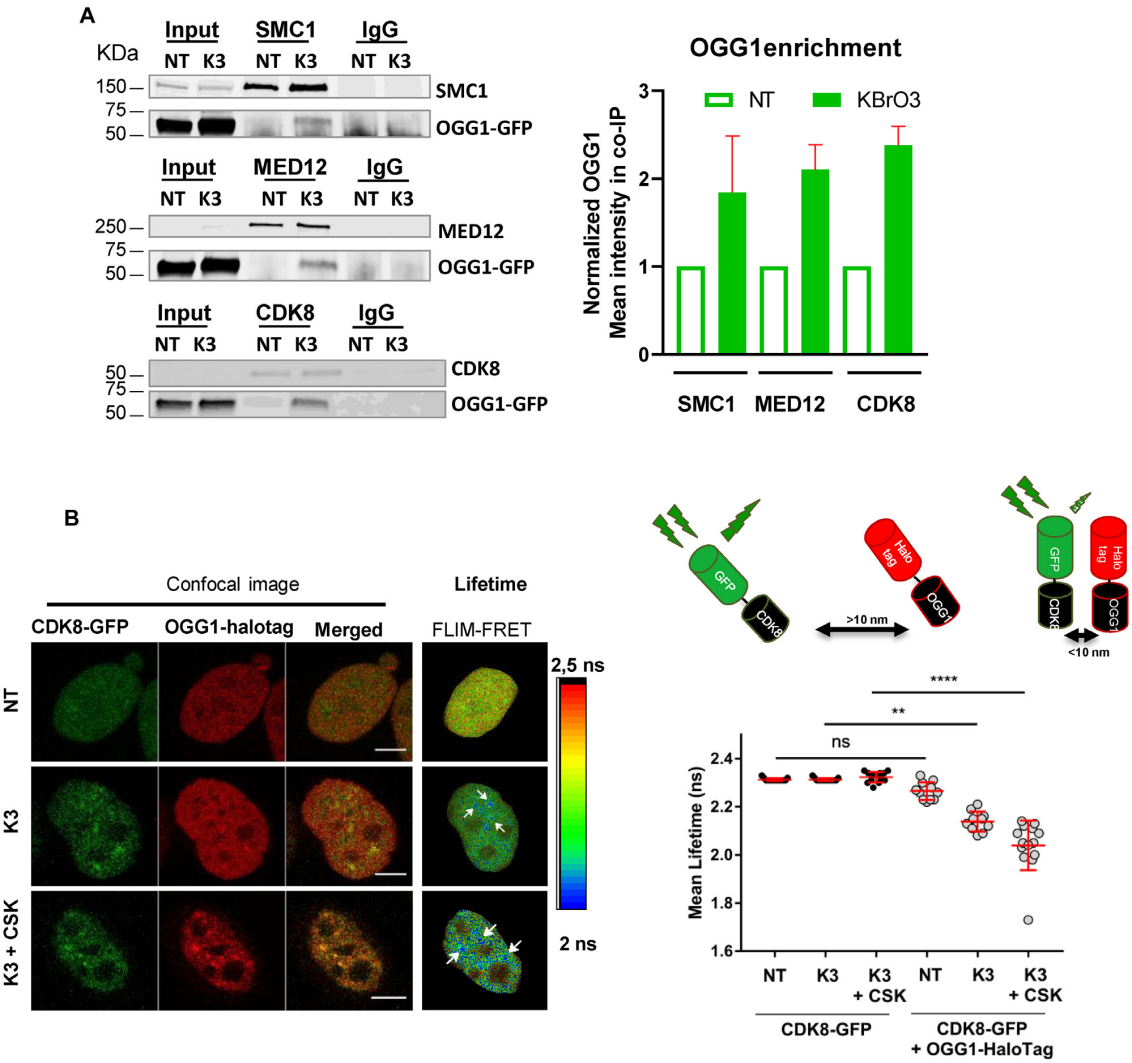
Oxidative stress induces the association of OGG1 with mediator and cohesins. (**A**) Immunoprecipitation of SMC1, MED12 and CDK8 was performed in non- (NT) or KBr03- (K3) treated HeLa cells expressing OGG1–GFP after benzonase treatment of the cell extracts. The presence of OGG1, SMC1, CDK8 and MED12 in different fractions (Input, IP using an antibody against the target protein or a control IgG) was evaluated by western blot using specific antibodies. Enrichment of OGG1 in the co-immunoprecipitates was quantified from 3 (MED12) or 2 (SMC1 and CDK8) independent experiments. Mean intensity measured was normalized to the amount detected in extracts from NT cells set to 1. Error bars represent SEM. (**B**) Intracellular distribution of OGG1-Halotag and CDK8-GFP fusion proteins in non- (NT) and KBrO_3_- (K3) treated HeLa cells. Prior to fixation, soluble proteins were removed with CSK as indicated. Scale bar: 5 μm. The spatial distribution of the mean fluorescence lifetime of the GFP donor is displayed using a continuous pseudocolor scale ranging from 2 to 2.5 ns. The graph corresponds to the quantification of donor's fluorescence lifetime in NT and K3 cells expressing the donor alone or the donor and the acceptor. Values obtained for 10 cells from a representative experiment are shown. The results were confirmed in three independent experiments and using mCherry as the acceptor instead of HaloTag (see Supplementary Figure S6). Statistical analysis involved a Kruskal–Wallis test. (****) *P* < 0.0001.

OGG1 co-localizes with mediator and cohesin in response to oxidative stress (Figure 4). To further investigate the potential association of OGG1 with those complexes, we immunoprecipitated the cohesin subunit SMC1 or the CKM mediator subunits MED12 and CDK8 to determine if OGG1 co-purified with them. OGG1 was not detectable when immunoprecipitations were performed in non-treated cells, but was observed in cells treated with KBrO_3_ (Figure 6A) suggesting that there is a physical association induced by oxidative stress between OGG1 and mediator and cohesin complexes.

In order to gain insight into the proximity between OGG1 and the CKM module after oxidative stress, we used a fluorescence lifetime imaging microscopy (FLIM) Förster's resonance energy transfer (FRET) assay. FLIM-FRET reflects the spatial distance between two molecules inside the cell based on the FRET between two fluorophores, which can only occur when they are less than 10 nm apart. This energy transfer results in a decrease in the fluorescence lifetime of the donor, measured by FLIM (25,26) (Figure 6B and Supplementary Figure S6). We first determined the fluorescence lifetime of the donor alone (CDK8-GFP), either in treated or non-treated cells, and either followed or not by CSK washes. We found no significant differences in fluorescence lifetimes between those three conditions, with a mean lifetime of 2.3 ns, corresponding to that of GFP (27). In cells expressing both the donor CDK8-GFP and the acceptor OGG1-Halotag, no significant alteration in GFP lifetime was observed in the absence of oxidative stress. However, in cells exposed to KBrO_3_ (K3) we observed a significant decrease in the GFP fluorescence lifetime. The CDK8-GFP lifetime was even shorter when determined in the chromatin fraction, after CSK washing (K3+CSK), suggesting that the association between the two proteins takes place principally on chromatin (Figure 6B and Supplementary Figure S6). The molecular proximity between CDK8 and OGG1 was independently confirmed by using OGG1-mCherry as an acceptor (Supplementary Figure S6).

### Mediator and cohesin are necessary for the presence of other BER factors on chromatin

**Figure 7.**
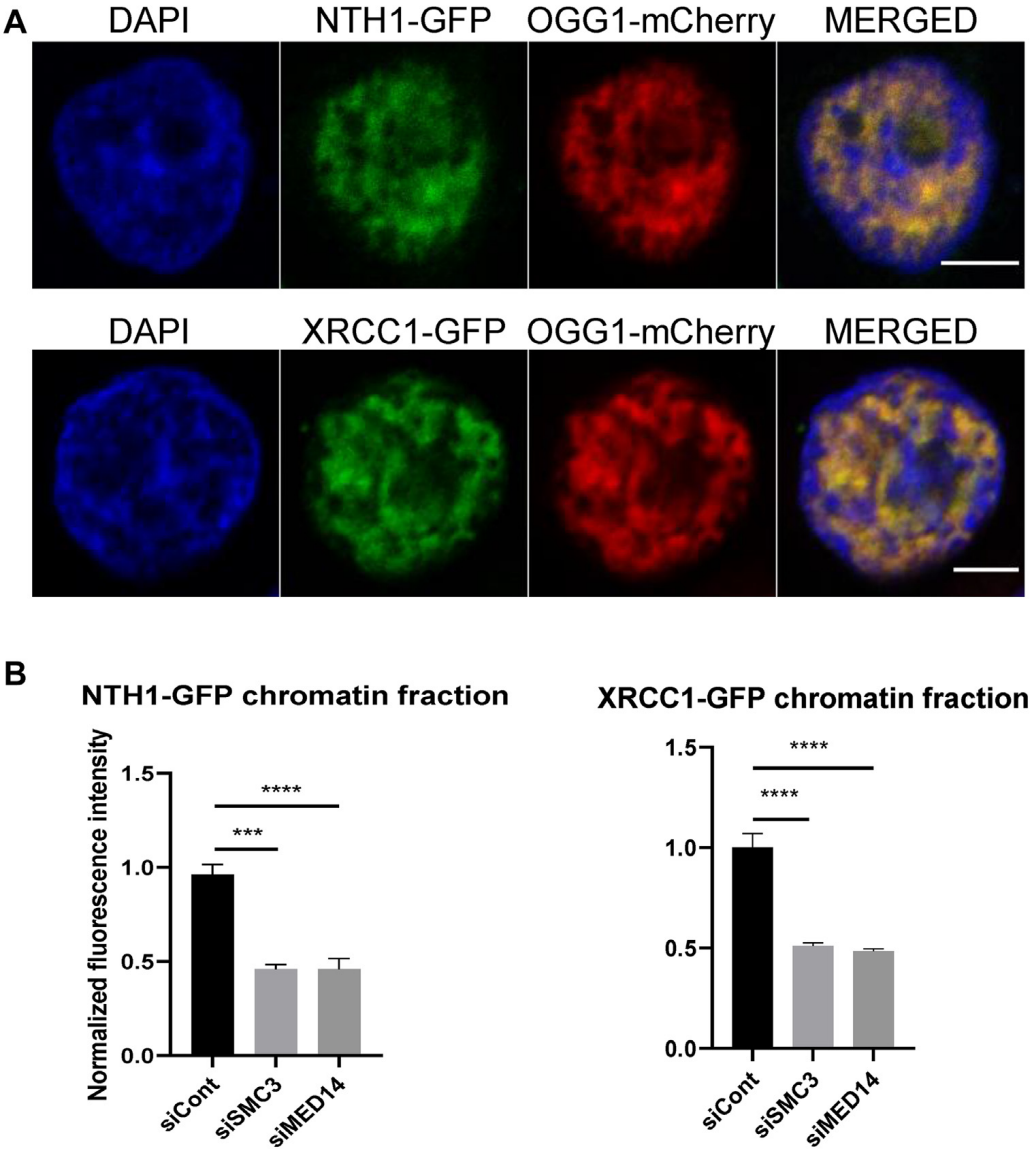
NTH1 and XRCC1 recruitment to chromatin after oxidative stress is dependent on mediator and cohesin. (**A**) Cells co-transfected with OGG1-mcherry and either NTH1-GFP or XRCC1-GFP were exposed to KBrO_3_ and after 3 h of recovery soluble proteins were washed by CSK pre-extraction. Both NTH1 and XRCC1 perfectly co-localize with OGG1 in the chromatin fraction. (**B**) Levels of NTH1-GFP and XRCC1-GFP associated to the chromatin fraction were quantified three hours after exposure to KBrO_3_ in cells transfected with siRNA against MED14 or SMC3 and compared to the control cells. More than 300 cells were analysed from two independent experiments. Error bars indicate SEM. Statistical analysis involved a Kruskal–Wallis test. (****) *P* < 0.0001; (***) 0.0005. Scale bar 5 μm

To determine if the requirements described above for OGG1 association with chromatin in response to an oxidative stress were extensible to other BER proteins, we tested first whether the recruitment to chromatin of a downstream factor in BER, XRCC1, was also dependent on the presence of MED14 and SMC3. The results shown in Figure 7 indicate that the subunits from both complexes, mediator and cohesin, are necessary for the association of XRCC1 with chromatin in response to oxidative stress. This result is consistent with the fact that the recruitment of XRCC1 to OGG1-initiated BER is through its interaction with the DNA glycosylase (6). Interestingly, NTH1, a DNA glycosylase initiating the repair of oxidized pyrimidines, is also re-localized to chromatin after oxidative stress in a MED14- and SMC3-dependent manner (Figure 7). These results suggest a general role for mediator and cohesin in the BER of oxidized bases.

## DISCUSSION

OGG1 can scan naked DNA with a high diffusion rate *in vitro* (2,28). However, the presence of reconstituted nucleosomes on an oligonucleotide harboring 8-oxoG inhibits OGG1 excision activity. This inhibition is alleviated *in vitro* by the addition of SWI/SNF chromatin remodelers (4), suggesting that other players involved in chromatin architecture and dynamics are required for the accessibility to and excision of 8-oxoG in the nuclear genome. Our previous studies have shown that the nuclear dynamics and distribution of OGG1 is affected by oxidative stress. OGG1 molecules that are freely diffusing in the nucleoplasm of untreated cells are retained in the chromatin fraction upon treatment with oxidizing agents such as KBrO_3_ or H_2_O_2_ (1,5) (Figures 1 and 2 and Figure S1).

Here, using a siRNA screen for factors involved in OGG1 search for its substrate, we have identified the cohesin and mediator complexes as being required for the association of OGG1 with chromatin. This suggests that the presence of 8-oxoG, which does not significantly distort the DNA double helix, is not sufficient to trigger the association of the DNA glycosylase with chromatin and, therefore, additional factors facilitate the process of detection and excision of the oxidized base (Figures 1-3). Indeed, the OGG1(F319A) mutant cannot recognize 8-oxoG and yet shows the same distribution pattern on chromatin as the WT protein, indicating that recognition of 8-oxoG by the DNA glycosylase does not drive recruitment of the protein (1). We show that induction of oxidative DNA damage results in the redistribution of the CDK8 and MED12 subunits of the mediator CKM module from the nucleoplasm to euchromatin, where they co-localize with OGG1. The physical association of OGG1 with mediator and cohesin complexes is required for chromatin recruitment or retention of the DNA glycosylase and thus for the processing of 8-oxoG lesions. Cohesin-binding sites are associated either with CTCF or with TFs, mediator and NIPBL (7). OGG1 and CTCF showed a mutually exclusive localization pattern of on chromatin after oxidative stress (Figure 4), whereas OGG1 both co-localized (Figure 4) and associated (Figure 6) with mediator subunits MED12 and CDK8. This result indicates that OGG1 is specifically retained at this second type of cohesin binding sites, and further suggests that cohesin and mediator complexes function as staging points throughout the genome to facilitate the repair of oxidative DNA damage.

Our study reveals that MED14, an essential subunit of the mediator core module (21), acts to recruit OGG1 to chromatin upon oxidative stress. We also identified the MED12 subunit as a hit in the screen, which forms part of the CKM module, together with the MED13, CDK8 and Cyclin C subunits. The kinase CDK8 and its paralog CDK19 are the only mediator subunits with enzymatic activity and both co-localize with OGG1 in the chromatin fraction of cells exposed to oxidative DNA damage. Although CDK8/19 enzymatic activity has been shown to be required for transcriptional regulation in several cases, many examples have shown a structural role of this subunit independent of its kinase activity (29). Here, we demonstrate that the requirement of CDK8 for OGG1 role involves its structural/scaffolding role rather than the kinase activity that we show is not essential for the chromatin retention of OGG1 nor for facilitating the excision of 8-oxoG (Figure 5). The CKM module is only transiently bound to the mediator complex (29) and up to ∼30% of the CKM sub-complexes are not associated with mediator (22) and may have an independent role. Our results demonstrate that not only MED12 and CDK8 subunits of CKM module but also the mediator core and MED13, that links the CKM module with the rest of mediator (13), are essential for OGG1 association with chromatin (Figures 1 and 5). This suggests that the role of the CKM subunits in BER unveiled in our study is not independent from the rest of the mediator complex.

Evidence suggests that 8-oxoG is not randomly distributed in the genome. Genome-wide profiling of 8-oxoG in rat kidney cells showed that genic regions have low levels of the lesion compared to gene deserts (30), suggesting that actively transcribed regions are preferentially repaired. This hypothesis is consistent with the recruitment of the BER machinery to euchromatin regions (1) and the association of OGG1 with cohesin and mediator, described here. Cohesins and mediator have been shown to be required for the formation of transcription factories and for TF access to DNA (31). Nucleosome depletion and clustering of TFs occurs in regions of the genome co-occupied by cohesin and mediator, and loss of cohesin decreases both TFs binding to clusters and DNA accessibility (14). Mediator can also contribute to SWI/SNF-dependent nucleosome displacement during transcription activation (32). It is thus tempting to speculate that the DNA accessibility associated with cohesin- and mediator-bound regions facilitates both the recruitment or retention of BER proteins and their access to the lesion.

Specific mechanisms exist to avoid interference between transcription and DNA repair (33), as in transcription-coupled nucleotide excision repair (TC-NER), where UV-induced DNA damage directly blocks the progression of RNA pol II and triggers the recruitment of the NER machinery (34). Although 8-oxoG by itself does not represent a block to the transcriptional machinery (35), it has been shown that repair intermediates generated by OGG1, such as abasic sites or single strand breaks, or the presence of the BER machinery itself, can interfere with transcription progression (36), suggesting that BER and transcription need to be coordinated. Here, we observed that cohesin and several of the mediator core subunits are constitutively associated with chromatin (Figure 4) whereas the CKM module subunits MED12 and CDK8 only associate with chromatin in cells exposed to oxidative stress, similarly to OGG1 (Figure 4). Several studies have shown a repression of transcription upon association of the CKM module with the rest of the mediator complex (37). It is therefore appealing to hypothesize that the recruitment of the CKM subunits is required not only to allow the loading of the DNA glycosylase onto DNA but also to repress transcription in order to avoid collisions/interference between the transcription machinery and the BER process. However, since mediator and cohesin defects have profound effects on gene expression, we cannot rule out that their association with OGG1 after an oxidative stress unveiled here could play other roles in addition to facilitate repair of oxidized bases. Indeed, OGG1 activity in G-rich regulatory elements of stress-responsive genes has been linked to the regulation of their expression (38).

Mediator and cohesin complexes have previously been linked to DNA repair. Cohesin is required for the repair of double strand breaks by homologous recombination in S and G2 phases of the cell cycle. It has been proposed to hold sister chromatids together through its cohesion function, thereby facilitating efficient repair (39–42) and preventing ectopic recombination (43). Our results indicate that the role of cohesin in BER is probably different, and independent of its cohesion function, as cohesin is required for OGG1 association with chromatin throughout the cell cycle (Supplementary Figure S2). Based on the interactions between mediator and NER factors, it has been proposed that mediator is involved in the DNA repair of UV induced lesions (44,45). Whether mediator's roles in NER and BER are the same remains to be determined.

The involvement of cohesin and mediator in the repair of DSBs, UV DNA-damage, and now in BER, suggests that those complexes may act as master regulators for the maintenance of genomic stability. Cohesin and mediator complexes could facilitate both the recruitment of DNA repair machineries to particular genomic locations, as well as chromatin accessibility. Inhibitors targeting BER proteins, such as APE1 and PARP1, are used in cancer therapy (46,47). The use of DNA glycosylase inhibitors may be limited by the considerable overlap in substrate specificity among the various glycosylases that could explain the weak phenotypes observed in mice when only a single enzyme is knocked out (48). Thus, the identification of general factors essential for BER could provide attractive targets for the development of cancer therapies.

## Supplementary Material

gkaa611_Supplemental_Files

## Notes

Present address: Emilie Lebraud, IRSN, F-92265 Fontenay aux Roses, France.
