# Supplementary material for: Chromatin recruitment of OGG1 requires cohesin and mediator and is essential for efficient 8-oxoG removal
Source: Nucleic Acids Res. 2020 Jul 25;48(16):9082–97. doi: 10.1093/nar/gkaa611 (PMC7498353; doi:10.1093/nar/gkaa611)

SUPPLEMENTARY INFORMATION

Supplementary methods

siRNA screening

To transfect HeLa cells with the individual siRNAs from the Qiagen Druggable siRNA library an automated reverse transfection protocol was developed on a robotic workstation equipped with a 96-well head probe (Nimbus, Hamilton). The siRNAs were lipoplexed with Lipofectamine RNAiMAX (Life Technologies) in collagen-coated, clear-bottom, black-walled 384-well culture plates (Greiner µClear plates, Cat# 781091). After 15 minutes of complexation, Hela-OGG1-GFP cells were seeded on top of the lipoplexes (1000 cells/well; final [siRNA] = 25nM), and incubated for three days at 37°C and 5% CO_2 in a humidified incubator. To minimize positional errors, each siRNA from the library was individually transfected as separate duplicate in different well positions of two independent culture plates. Each culture plate also received an identical set of positive and negative controls: transfection reagent alone (12 "MOCK" wells, negative control); an irrelevant siRNA (28 "UNR" wells, negative and normalization control, target sequence: AAGCCGGTATGCCGGTTAAGT, Qiagen) ; a pool of cytotoxic siRNAs (8 "AllStars" wells, transfection positive control, Allstars maximal death control, Qiagen); an siRNA against EGFP (4 "GFP Control" wells, positive control, SI04379956, Qiagen); and an siRNA against hOGG1 (6 "siOGG1" wells, positive control, target sequence: GGAUCAAGUAUGGACACUGA, Qiagen). Three days post-transfection, culture supernatants were removed using an automated plate washer (ELX405, Biotek), and oxidative DNA damage were induced by exposing cells to $KBrO_3$ in 40 mM in PBS for 70 min at 37°C. The $KBrO_3$ solution was then removed by aspiration, replaced with warm, complete culture medium, and plates were incubated for 3h30 at 37°C. Cells were then pre-extracted in ice-cold Cytoskeleton Buffer (CSK Buffer: PIPES pH 6.8 10mM, NaCl 100mM, Sucrose 300mM, $MgCl_2$ 3mM,Triton-X100 0.5% [v/v], EGTA pH 8 1mM in H_2O) during 8 min to remove the soluble protein fraction, including the free form of OGG1-GFP. Plates were then fixed overnight with para-formaldehyde (2% [w/v] final in PBS, Sigma), and nucleic acids were stained with Hoechst 33342 (1.25µg/mL final, Sigma). After a PBS wash, plates were imaged on a High Content Imaging device (Operetta HCS epifluorescence microscope, Perkin Elmer). Three fields per well were acquired at 10X magnification, in two fluorescence channels: green for GFP (ex: 470±10nm; em: 525±25nm) and blue for Hoechst 33342 (ex: 380±20nm; em: 445±35nm). An automated algorithm was developed under Harmony 3.0 (Perkin Elmer) to quantify 3 main parameters: the Total cell amount, the percentage of GFP positive cells (%GFP+ cells) and the average Nuclear Specific GFP fluorescence (NSF). Briefly, nuclear Regions of Interest (ROI) were segmented in the Hoechst channel and used to quantify the

Total cell amount per well. Cells were then defined as GFP positive when their average nuclear GFP signal was found 3 times above the GFP background fluorescence (as measured in the green channel, in the immediate surroundings of the nuclear ROIs). The %GFP+ cells values were then computed as the proportion of GFP+ cells relative to the Total cell amount. Finally, NSF values were computed from the subpopulation of Hela cells expressing OGG1-GFP, as the average nuclear GFP signal corrected by the GFP background.

Data preprocessing was performed in R (R Foundation for Statistical Computing, Vienna, Austria. https://www.R-project.org/). For each culture plate, the Total cell amount, the %GFP+ and the NSF values measured in sample wells were first normalized to the averaged values measured in their respective negative control (UNR) wells. Normalized results were labeled as Relative Total Cell amount, Relative %GFP+ and Relative NSF. We then analyzed if the screening technical processes could have introduced some systematic bias in our data. To achieve this, we computed the averaged Relative NSF and Relative %GFP+ from the sample values at each well index (A1, B1, C1 etc..). The results were plotted as 3D model estimators of topological trends in 384-well format. We noticed some edge effects in both Relative NSF and Relative %GFP+ measurements, that were corrected using an adaptation of the method described by (1). Briefly, we used a simple non-parametric loess regression fit to determine, for a given well index, the best fitted value between a well value (Relative NSF or Relative %GFP+) and its corresponding plate index. Then, for a given plate and well index, a specific offset was computed as the delta of its corresponding fitted well value over the median of the screen sample values. These offsets were then subtracted from each individual values at their corresponding plate and well indexes. The corrected results were labelled as Corrected Relative NSF and Corrected Relative %GFP+.

Corrected Relative NSF and Corrected Relative %GFP+ sample results were analyzed using two concurrent scoring methods. We first applied, plate-wise, a robust Z-Score to the Corrected Relative NSF and Corrected Relative %GFP+ sample values. Briefly, this sample-based scoring method rescales the plate data in a way that the sample distributions have a median of 0 and a median average deviation of 1.

We also applied a B-Score (2) metric to our data. Briefly, B-Score is a sample-based scoring method first relying on the iterative application of a two-way median polish algorithm to correct plate values for systematic positional effects. Because this method includes a form of topological correction, B-Score was applied on the uncorrected Relative %GFP+ and Relative NSF values. The algorithm then transforms each residual well value into a score indicating its deviation - in terms of Median Average Deviations - from the median of the plate residuals population.

As a result, Robust Z-Score and B-Scores above 2 and 3 correspond to samples statistically deviating from the population of inactive samples, with associated confidence intervals above 0.95 and 0.99, respectively.

We choose a very conservative approach to select our candidate genes. An siRNA was considered as "active" when at least one of its NSF or %GFP+ replicate value was found with either a Z-Score or B-Score above 2 (Moderate activity) or 3 (High activity). siRNAs with positive and negative scores were labeled as "Stimulators" and "Inhibitors", respectively. For a given parameter (NSF or %GFP+), a gene was defined as a candidate either when at least 2 out of the 3 tested siRNAs had High activity, or when the 3 tested siRNAs had Moderate activity, always in the same direction (Stimulators or Inhibitors). Where also considered as candidates genes for which 2 out of 3 tested siRNAs deviated of 3SD, or when the 3 tested siRNAs deviated of 2SD, from the UNR negative controls population.

The candidate genes were retested in a second round of screening with 4 siRNAs per gene (3 siRNAs originally tested in the primary screen plus an additional one). These siRNAs were cherry-picked from the Druggable siRNA library using an 8-channel automated workstation (Star, Hamilton), and tested in triplicates in the same cell-based assay as used for Primary Screen. To eliminate potential false positives originating from unspecific siRNA effect on hOGG1-GFP expression, a concurrent secondary screen was also performed in a similar cell-based assay, but without CSK treatment ("Control screen").

As for the Primary screen, Total cell amount, %GFP+ cells and NSF sample data from each culture plates of the secondary screen were normalized to the averaged values measured in their respective negative control (UNR) wells. These normalized results were labelled, as before, as Relative Total Cell amount, Relative %GFP+ and Relative NSF. To correct for siRNA unspecific effects on OGG1-GFP expression, we computed the ratio of the Relative %GFP+ and Relative NSF data from the $KBrO_3$ & CSK treated plates over the corresponding normalized data from the Control screen. The obtained data were labelled as Relative %GFP+ ratio and Relative NSF Ratio. An siRNA was defined as active when its averaged Relative %GFP+ ratio or its averaged Relative NSF Ratio value was found deviating from at least 2.SD above (=Stimulator) or below (=Inhibitor) the averaged negative controls value. A gene was selected as hit when at least three siRNAs out of the four tested were found active in the same direction on Relative NSF Ratio or Relative %GFP+ ratio. A hit gene with three or four active siRNAs was classified as "High Confidence Hit" or "Very High Confidence Hit", respectively. Additionally, a hit gene was classified as "Cytotoxic/cytostatic" when at least three out of four tested siRNAs had an averaged Relative Total Cell amount value equal or below 3.SD from the averaged negative controls value.

Results of the primary and secondary screens are presented in Supplementary Table S1.

Alkaline elution

The alkaline elution assay was used for the quantification of SSB and FPG-sensitive DNA modifications in the chromosomal DNA (3). The assay makes use of the facts that (i) Fpg protein converts its substrate modifications into SSB, and that (ii) the elution rate of the DNA from a membrane filter depends on the average length of the DNA molecules, i.e. on the number of SSB in the DNA. Briefly, 10^6 cells were lysed on the filter (polycarbonate; 2 µm pore size; 25 mm diameter) by incubation with a lysis solution (100 mM glycine, 20 mM Na_2EDTA, 2% SDS, 400 µg/ml proteinase K, pH 10.0) for 90 min at 25°C. After extensive washing (20 mM Tris/HCl, 100 mM NaCl, 1 mM Na2EDTA, pH 7.5), the DNA remaining on the filter was incubated for 60 min at 37°C with FPG protein (1 µg/ml in 20 mM Tris/HCl, 100 mM NaCl, 1 mM Na_2EDTA, pH 7.5), immediately before elution from the filter with an alkaline solution [20 mM EDTA (acid form) adjusted to pH 12.15 with tetraethylammonium hydroxide] at 25°C. The incision by the enzyme at its substrate modifications was shown to be saturated under these conditions. The percentage of DNA remaining on the filter after various elution times (up to 10 h) was determined by fluorescence measurements after neutralization, using the dye Hoechst 33258 (final concentration 0.75 µM). The numbers of SSB plus FPG-sensitive modifications were calculated from the slopes of the elution curves. To quantify SSB, the incubation was carried out on filters without the repair enzyme in parallel; this allows to calculate the number of FPG-sensitive modifications by subtraction. For calibration, γ-irradiated cells were used, assuming that 6 Gy generate 1 SSB per 10^6 bp. For the measurements of the repair kinetics of the lesions induced by $KBrO_3$, the number of DNA modifications observed in untreated control cells (basal damage levels) were subtracted.

HPLC-MS/MS Analysis

DNA extraction was performed using the ‘chaotropic’ method that minimizes DNA oxidation during the work-up. Briefly, 750 µl of lysis buffer A (320 mM sucrose, 5 mM MgCl2, 10 mM Tris, 0.1 mM defferroxamine pH 7.5, 1% Triton X-100) was added to the cellular pellet. After a vigorous agitation, nuclei were collected by centrifugation at 1500 g for 10 min at 4°C and washed with 750 µl of buffer A. To the nuclear pellet, obtained after centrifugation (1500 g for 10 min at 4°C) was added 300 µl of buffer B (10 mM Tris, 5 mM EDTA-Na2, 0.15 mM defferroxamine, pH 8.0) and 18 µl of SDS 10%. A vigorous agitation was performed to allow lysis of the nuclear membrane. Thereafter, 1.5 µl of RNase A (100 mg/ml) in RNase buffer (10 mM Tris, 1 mM EDTA, 2.5 mM defferroxamine, pH 7.4) and 3.5 µl of RNase T1 (1 U/µl in RNase buffer) was added and the samples were incubated for 15 min at 50°C. Then, 15 µl of Qiagen protease (20 mg/ml in H_2O) was added prior to incubation at 37°C for 1 h. Subsequently, 0.6 ml of the NaI solution (7.6 M NaI, 40 mM Tris, 20 mM EDTA-Na_2, 0.3 mM

defferroxamine, pH 8.0) and 0.5 ml of 2-propanol was added. DNA precipitation was achieved by gently inverting the tube several times. DNA was recovered by centrifugation at 5000 g for 15 min at 4°C and washed with 500 µl of 40% 2-propanol. After centrifugation (5000 g for 15 min), DNA was washed again with 500 µl of 70% ethanol. DNA was recovered by centrifugation and dissolved into 50 µl 0.1 mM deferoxamine containing 0.1 µM 8-oxodGuo-$^{15}N_5$. DNA digestion was performed by adding 0.25 µL of phosphodiesterase II, 0.25 µL of DNase II, 2.5 µL of nuclease P1 buffer (300 mM ammonium acetate and 1 mM $ZnSO_4$, pH 5.3), and 2.5 µL of buffer MNSPDE (200 mM succinic acid, 100 mM $CaCl_2$, pH 6). The samples were incubated for 2 h at 37 °C. Then, 6 µL of buffer Palk 10× (500 mM Tris and 1 mM EDTA, pH 8), 0.5 µL of phosphodiesterase I and 2 U of alkaline phosphatase were added. The resulting solutions were incubated again for 2 h at 37°C. Finally, 3.5 µL of hydrochloric acid 0.1 N was added. The resulting nucleoside mixture was then analyzed by HPLC-MS/MS.

Liquid chromatography HPLC-MS/MS analysis was performed using an Accela system (Thermo Electro Corporation). The separation of nucleosides was carried out on an ODB uptisphere column (150 × 2.1 mm 3 µm) from Interchim under linear gradient conditions as previously reported (4). The temperature of the column was maintained at 28°C. The flow rate was set at 0.2 mL/min with 2mM ammonium formate as the initial mobile phase and then a gradient of CH_3OH from 0 to 30% over 30 min was used. The MS/MS detection was performed with a triple quadrupole mass spectrometer TSQ Quantum Ultra from Thermo Scientific equipped with a Thermo Accela Pump, an Accela autosampler and an Accela photodiode array detector. The electrospray ionization interface (ESI) source was operated in the positive ionization mode. The multiple reactions monitoring mode (MRM) was used in order to specifically detect 8-oxoG and its isotopically labeled internal standard. 8-oxoG and 8-oxoG $^{15}N_5$ were detected using transitions 284 → 168 and 289 → 173, respectively, and eluded 11.8 min. All data collected in centroid mode were processed using Xcalibur software. Unmodified nucleosides were detected by UV absorption and quantification was performed by external calibration. The number of DNA modifications observed in untreated control cells (basal damage levels) were subtracted.

Supplementary references

1. Kevorkov,D. and Makarenkov,V. (2005) Quality control and data correction in high-throughput screening. *J. Biomol. Screen. Off. J. Soc. Biomol. Screen.*, **10**, 1–5.
2. Brideau,C., Gunter,B., Pikounis,B. and Liaw,A. (2003) Improved statistical methods for hit selection in high-throughput screening. *J. Biomol. Screen.*, **8**, 634–647.
3. Pflaum,M., Will,O. and Epe,B. (1997) Determination of steady-state levels of oxidative DNA base modifications in mammalian cells by means of repair endonucleases. *Carcinogenesis*, **18**, 2225–2231.
4. Ravanat,J.-L., Douki,T., Duez,P., Gremaud,E., Herbert,K., Hofer,T., Lasserre,L., Saint-Pierre,C., Favier,A. and Cadet,J. (2002) Cellular background level of 8-oxo-7,8-dihydro-2'-deoxyguanosine: an isotope based method to evaluate artefactual oxidation of DNA during its extraction and subsequent work-up. *Carcinogenesis*, **23**, 1911–1918.

Supplementary Table S1. Results of the primary and secondary siRNA screens. See Supplementary methods for details concerning description and analysis. Results obtained for cohesin subunits RAD21, SMC3 and SMC1 and Mediator subunits MED12 and MED14 have been highlighted.

(Excel file uploaded as a separate file)

Supplementary Table S2. List of plasmids used in this study.

**Plasmids**	**SOURCE**	**IDENTIFIER**
OGG1-GFP	Campalans et al., 2013	N/A
NTH1-GFP	Campalans et al., 2013	N/A
XRCC1-GFP	Campalans et al., 2013	N/A
OGG1-Dendra2	This paper	Described in Method details (construction of plasmids)
OGG1-mCherry	This paper	Described in Method details (construction of plasmids)
OGG1-HaloTag	This paper	Described in Method details (construction of plasmids)
CDK8-GFP	This paper	Described in Method details (construction of plasmids)
CDK19-HaloTag	PROMEGA	Cat# FHC01935
MED12-HaloTag	PROMEGA	Cat# FHC12080

Supplementary Table S3. Sequences of oligonucleotides (for plasmid construction) and siRNA (for silencing of indicated targets) used in this study.

**Oligonucleotides (sequences 5' -> 3')**	**SOURCE**	**IDENTIFIER**
siRNA control	QIAGEN	SI03650318
siRNA MED12	QIAGEN	SI00072660<br>CACCCAAACCCTGGTTCTATA
siRNA MED13	QIAGEN	SI03083577<br>CCTGTCGATTAGGTCAACATA
siRNA CDK8	QIAGEN	SI00024402<br>TTCGAGAGCTTAAGCATCCAA
siRNA cyclin C	QIAGEN	SI00073507<br>CAAGTACCGTATATTGATTTA
siRNA MED14	QIAGEN	SI00061327<br>CGGGTGAAGTTTCGTGTTGAA
siRNA SMC3	QIAGEN	SI02654281<br>GAGGACTAAGTTGGAGCTTAA
siRNA RAD21	QIAGEN	SI00086688<br>AACACTGAAAGTTCTAGCTTA
siRNA SMC1A	QIAGEN	SI00087248<br>CGGCGTATTGATGAAATCAAT
SP0054	Eurogentec custom	TCCGGAGCGGCCGCTGCAGGAGGCAGCCA<br>AAAAATGGTGAGCAAGGGCG
SP0055	Eurogentec custom	GCCGCTCCGGATGGATCCGGGCCTTC
SP0475	Eurogentec custom	GTTCCAAAGGGCCGGAAGGCCCGGATCCAT<br>CCGGAGCCGCGGCTGCAGGAGGCAGCTCC<br>GGAGCCGCGGCTGCAGGAGGCAGC
SP0476	Eurogentec custom	GCCTCCTGCAGCGGCCGCTCCGGAGCTGCC<br>TCCTGCAGCCGCGGCTCCGGAGCTGCCTCC<br>TGCAGCCGCGGCTCCGGATG
SP0674	Eurogentec custom	AGCGGCCGCGAC
SP0675	Eurogentec custom	CCTTGCTCACCATTTTTTG
SP0676	Eurogentec custom	CTGCAGGAGGCAGCCAAAAAGAAATCGGTA<br>CTGGCTTTCC
SP0677	Eurogentec custom	ATCTAGAGTCGCGGCCGCTCTATTAACCGGA<br>AATCTCCAGAGTAGACAGC
SP0584	Eurogentec custom	GATCTCGAGCTCAAGCTTCGAATTCGCCACC<br>ATGGACTATGACTTTAAAGTGAAGC
SP0585	Eurogentec custom	CACCATGGTGGCGACCGGTGGATCCCCGTA<br>CCGATGTGTCTGATGTG
SP0625	Eurogentec custom	ACCGGTCGCCACC
SP0626	Eurogentec custom	GAAGCTTGAGCTCGAGATC

Supplementary Table S4. List of antibodies used in Western Blot and immunofluorescence experiments

Antibody	SOURCE	IDENTIFIER
Normal rabbit IgG	Santa-Cruz Biotechnology Inc	Cat# SC-2027
8-oxoguanine	Abcam	Cat# ab48508, RRID:AB_867461
SMC3	Abcam	Cat# ab128919, RRID : AB_11150430
CTCF	Abcam	Cat# ab128873, RRID : AB_11144295
CENP-F	Abcam	Cat# ab5, RRID : AB_304721
GFP	Abcam	Cat# ab290, RRID : AB_303395
OGG1	Abcam	Cat# ab124741, RRID : 10973360
RAD21	Abcam	Cat# ab992, RRID : AB_2176601
SMC1	Abcam	Cat# ab21583, RRID : AB_2192477
Lamin B1	Abcam	Cat# ab20396, RRID : AB_445561
MED14	Abcam	Cat# ab72141, RRID : 1209425
MED12	Abcam	Cat# ab70842, RRID : AB_1269392
MED13	Abcam	Cat# ab49468, RRID : AB_1280997
MED17	Thermo Fisher Scientific	Cat# PA5-30314
CDK8	Santa-cruz	Cat# Sc-13155, RRID : AB_627244
Cycline C	Abcam	Cat# ab85927, RRID : AB_1924908
STAT1 phospho-S727	Cell Signaling Technology	Cat# 9177S, RRID : AB_2197983
HP1α	Euromedex	Cat# 2Hp-2G9-AS
H3	Active Motif	Cat# 39763, RRID : AB_2560522
Anti-mouse IR800	Advansta	Cat# R-05061-250
Anti-mouse IR700	Advansta	Cat# R-05055-250
Anti-rabbit IR800	Advansta	Cat# R-05060-250
Anti-rabbit IR700	Advansta	Cat# R-05054-250
Anti-mouse Alexa 488	Thermo Fisher Scientific	Cat# A11001, RRID : AB_2534069
Anti-rabbit Alexa 594	Thermo Fisher Scientific	Cat# A11012, RRID : AB_2534079

Supplementary Table S5. List of reagents used in this study

Reagents	SOURCE	IDENTIFIER
Potassium bromate	Sigma	Cat# 60085
Hydrogen peroxide	Sigma	Cat# 95321
G418	Gibco	Cat# 10131-027
DMEM	Gibco	Cat# 31966-021
FBS	Sigma	Cat# F7524
EcoRI	New England Biolabs	Cat# R0101S
BamHI	New England Biolabs	Cat# R0136S
DpnI	New England Biolabs	Cat# R0176S
T4 DNA polymerase	New England Biolabs	Cat# M0203S
Phusion DNA polymerase	New England Biolabs	Cat# M0530S
Fast-AP	ThermoFisher Scientific	Cat# EF0654
DAPI	Molecular Probes	Cat# D1306
RNAse A	Sigma	Cat# R5500 Cat# 28908
Formaldehyde	ThermoFisher Scientific	Cat# 28908
Paraformaldehyde	SIGMA	Cat# 158127
Hoechst 33342	SIGMA	Cat# B2261
LipoFectamine 2000	Thermo Fisher Scientific	Cat# 11668-019
LipoFectamine RNAiMAX	Thermo Fisher Scientific	Cat# 13778-150
Fluorescence mounting medium	Dako	Cat# S3023
Benzonase	Merck Millipore	Cat# 70664
GeneJet PCR purification kit	(Thermofisher)	Cat# K0701
4-15% Mini-PROTEAN TGX Precast Protein Gels	Biorad	Cat# 456-8083
Trans-Blot Turbo mini Nitrocellulose membranes	Biorad	Cat# 170-4158
Trans-Blot Turbo midi Nitrocellulose membranes	Biorad	Cat# 170-4159
Dynabeads	Invitrogen	Cat# 11204D
Halotag TMR ligand	Promega	Cat# G8251
Cortistatin A	From Henry Pelish and Matthew Shair	Lee et al., 2008

Supplementary Table S6. List of softwares used in this study

**Softwares**		
GraphPad prism7	GraphPad software	https://www.graphpad.com/scientific-software/prism/
ImageJ version 1.51 j8	NIH, USA	https://imagej.nih.gov/ij
NIS-elements	Nikon Instruments	https://www.nikoninstruments.com/fr_FR/Produits/Logiciels/NIS-Elements-Confocal
Image Studio	Odyssey software	
ImageQuant	GE Healthcare	
SymPhoTime	Leica software	
Harmony 3.0	Perkin Elmer	http://www.perkinelmer.com/fr/product/harmony-4-8-office-hh17000001
R	R Foundation for Statistical Computing, Vienna, Austria	https://www.R-project.org/
Lasergene Structural Biology Suite – version 14.1.0	DNA Star	https://www.dnastar.com

Supplementary Figure legends

Figure S1: Cohesin and Mediator complexes are essential for the association of OGG1 with chromatin after induction of oxidative DNA damage.

(A) Gene-knock down effect on OGG1-GFP association to chromatin was assessed by measuring the relative Nuclear Specific GFP Fluorescence (NSF) and the relative amount of GFP positive cells (%GFP+) as described in Supplementary Methods. For each of the three tested set of siRNAs (labelled as Sets A, B, and C) averaged NSF duplicate results were plotted against the averaged %GFP+ cells duplicate results. siRNAs targeting the selected candidate genes are highlighted in red for GFP and/or NSF inhibitors, and in green for GFP and/or NFS stimulators. siRNAs targeting MED12, MED14, SMC1A, SMC3 and RAD21 are highlighted as black dots in each set of data set. The siRNA labelled in blue are considered as inactive.

(B) OGG1-GFP association with chromatin induced by $KBrO_3$ is impaired in cells depleted for cohesin subunits SMC1 and RAD21. Hela cells stably expressing OGG1-GFP were transfected with siRNAs against SMC1 and RAD21 and exposed to $KBr0_3$. Three hours after treatment cells were pre-extracted with CSK (to remove the soluble fraction) and fixed and nuclei stained with DAPI. Quantifications are presented in Figure 1. The efficiency of siRNAs targeting SMC1 and RAD21 was evaluated by Western blot (right panel)

(C) OGG1-GFP association with chromatin is impaired in cells depleted of Mediator and cohesin complexes. The presence of OGG1-GFP at the chromatin fraction 6 and 9 hours after $KBrO_3$ exposure was quantified after removal of the soluble fraction with CSK pre-extraction. Around 1000 cells from two independent experiments were analysed for each siRNA. Values were normalized to the ones obtained 6 hours after $KBrO_3$ exposure in cells transfected with the siRNA control were set to 1. Results are expressed as the mean GFP fluorescence +/- SEM. Statistical analysis was performed using a Kruskal-Wallis test. (****) $P < 0.0001$.

(D) Association of OGG1-GFP with the chromatin fraction induced by treatment with 1 mM H_2O_2 is impaired by the depletion of the Mediator subunits MED12 and MED14 and the cohesin subunit SMC3. More than 2000 cells from at least three independent experiments were analyzed as above. (****) $P < 0.0001$.

Figure S2: Mediator and cohesin are required for chromatin association of OGG1 throughout the cell cycle.

(A) OGG1 recruitment (green) in Hela OGG1-GFP expressing cells after $KBrO_3$ treatment and CSK washing can be observed in G1 (negative for both CENPF and EdU; green), S (positive for EdU; magenta) and G2 (positive for CENPF; red) phases of the cell cycle. Scale bar: 10 µm.

(B) Higher magnification of cells in the different phases of the cell cycle. Staining as in A. Scale bar: 5 µm

(C) Indicated cohesin and Mediator subunits were depleted in Hela cells stably expressing OGG1-GFP cells. Quantification of OGG1-GFP in the insoluble fraction (+ CSK) of non-treated (NT) cells and 3 hours after exposure to $KBrO_3$ (K3) was performed for the different cell cycle phases. Values represent mean with SEM. For each siRNA, at least 50 cells from three

independent experiments were analysed. Results obtained for each siRNAs were normalised to the control (siControl, K3 + CSK) for G1 cells. For statistical analysis, a Kruskal-Wallis test was performed. (****) $P < 0.0001$.

Figure S3: Recovery of the mobility of photoconvertible OGG1-Dendra2 in cells exposed to $KBrO_3$ treatment.

Protein dynamics of photoconvertible OGG1-Dendra2 in non-treated cells (NT) and 22 hours after exposure to $KBrO_3$. OGG1-Dendra2 was photoconverted from green to red using the 405 nm laser and both green and red signals were followed over time. Images obtained before photoconversion or at different times after photoconversion are shown. Quantification of the red signal in the photoconverted region was normalised to the red intensity immediately after photoconversion and displayed in the graph.

Figure S4: Validation of the immunofluorescence approach for the quantification of 8-oxoG.

(A) HeLa cells expressing OGG1-GFP were exposed to $KBrO_3$ and recovered 0, 3 or 6 hours after the treatment. The amount of unrepaired 8-oxoG was measured by three independent techniques: HPLC/MS-MS, Alkaline Elution and immunofluorescence. Results obtained from three independent experiments were normalized to the levels of damage measured at 0 Hours time point set to 100. Error bars represend SD.

(B) Excision of $KBrO_3$-induced 8-oxoG was measured in WT HeLa cells and in cells overexpressing OGG1-GFP at 2, 4 and 6 hours after treatment by HPLC/MS-MS. Results were normalized to the levels measured immediately after the treatment set to 100%. Mean +/- SEM of 8-oxoG levels measured from two independent experiments are shown.

(C) HeLa cells expressing different levels of OGG1-GFP were fixed three hours after exposure to $KBrO_3$ and stained with antibodies against 8-oxoG (green) and GFP (magenta). Nuclear DNA was stained with Propidium iodide (PI, red). 8-oxoG signal was quantified in cells expressing lower or higher levels of OGG1. A representative experiment out of three independent ones is shown, more than 350 cells were analysed. For statistical analysis, a Mann-Whitney test was performed. (****) $P < 0.0001$.

(D) 8-oxoG excision is impaired in cells depleted for Mediator or cohesin. Relative 8-oxoG levels were determined by immunofluorescence using an antibody against the lesion before and at different times after exposure to $KBrO_3$ in control cells or cells depleted for SMC3 or MED14. NT = non-treated, K0H, K4H and K8H = immediately, 4h and 8h after treatment, respectively. More than 1500 cells were analysed from three independent experiments. Mean Fluorescence intensity has been normalized to the levels detected in the siControl set to 1 at the different time points. Statistical analysis used a Kruskal-Wallis test. (****) $P < 0.0001$.

Figure S5: Behaviour of Mediator subunits upon exposure to oxidative stress.

(A) Distribution patterns of OGG1-Halotag and MED17 in non- (NT) and KBr03 (K3)- treated cells. Prior to fixation, soluble proteins were removed with CSK when indicated.

(B) Effect of depletion of MED14 on the presence of MED12 in the chromatin fraction of untreated cells (NT) and cells exposed to $KBrO_3$ (K) was evaluated by Western blot. Histone H3 was used as a loading control. The graph represents quantification for three independent experiments. Enrichment of the indicated proteins induced by $KBrO_3$ is represented as a ratio

of the levels measured in $KBrO_3$ compared to NT cells. Ratios are normalized to the values obtained in the cells transfected with the siControl set to 1. Error bars indicate SD.

Figure S6: Molecular proximity between OGG1 and the Mediator subunit CDK8 in cells exposed to oxidative stress.

(**A**) Illustration of intracellular distribution of CDK8-GFP and OGG1-mCherry in non- (NT) or $KBrO_3$- (K3) treated cells expressing only CDK8 or both CDK8 and OGG1. Scale bar: 5 µm. The spatial distribution of the mean fluorescence lifetime of the GFP donor is displayed using a continuous pseudocolor scale ranging from 2 to 2.5 ns. Graphs show the fluorescence decay of CDK8-GFP donor in cells expressing the donor alone or the donor and the acceptor OGG1-mCherry. More than 10 cells were analysed from two independent experiments. Statistical analysis was performed using a Mann-Whitney test. (****) $P < 0.0001$.

(**B**) Magnification of fluorescence lifetime images corresponding to cells exposed to $KBrO_3$ (presented in Figure 6B and S7A) and expressing the indicated fluorescent proteins. The spatial distribution of the mean fluorescence lifetime of the GFP donor is displayed using a continuous pseudo-colour scale ranging from 2 to 2.5 ns. Arrows point to the subnuclear regions where donor lifetime is the lowest (appearing in blue) indicating a molecular proximity closer than 10 nm between OGG1 and CDK8.

Figure S1: Cohesin and Mediator complexes are essential for the association of OGG1 with chromatin after induction of oxidative DNA damage

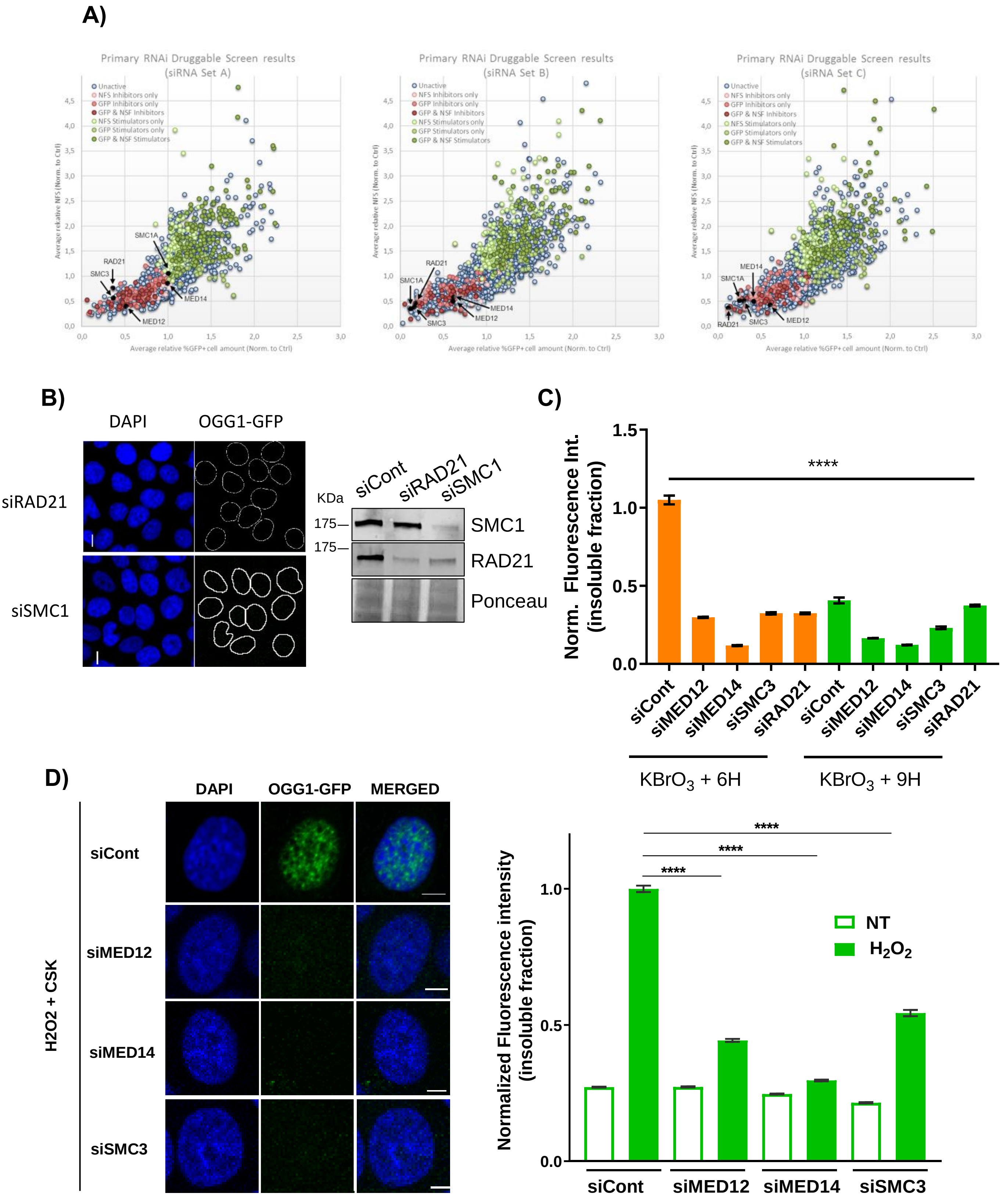

Figure S2: Mediator and cohesin are required for chromatin association of OGG1 throughout the cell cycle

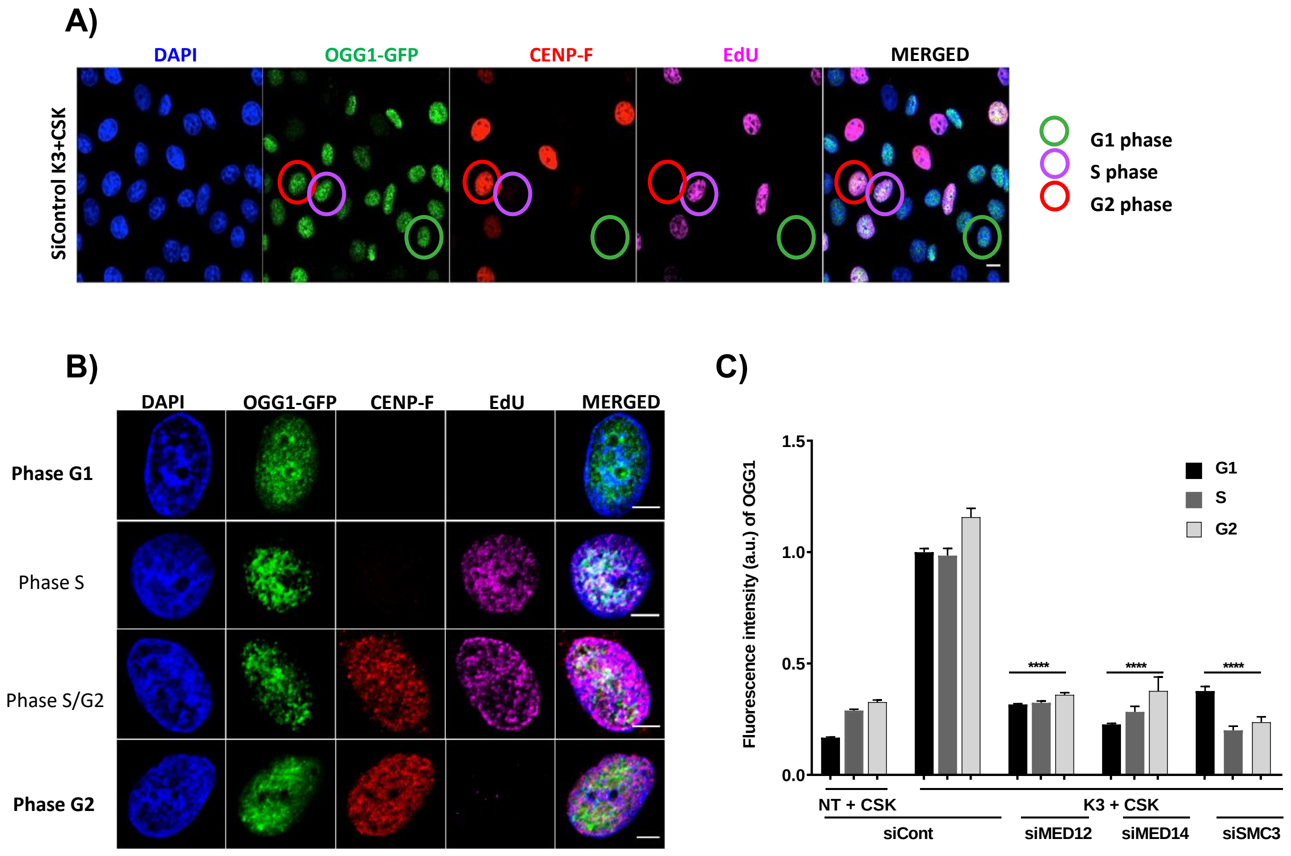

Figure S3: **Recovery of the mobility of photoconvertible OGG1-Dendra2 in cells exposed to KBrO3 treatment**

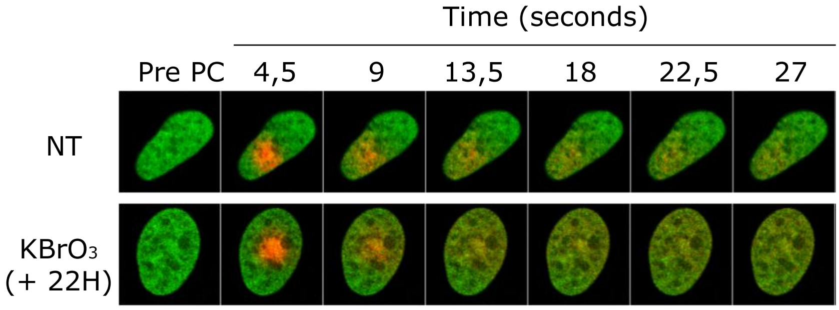

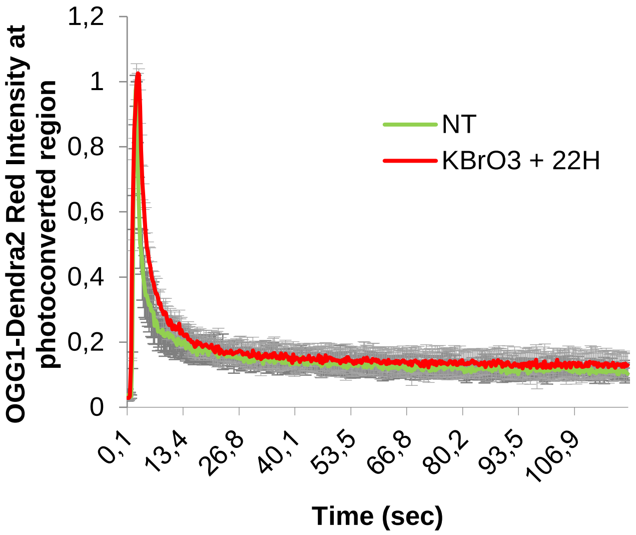

Figure S4: Validation of the immunofluorescence approach for the quantification of 8-oxoG

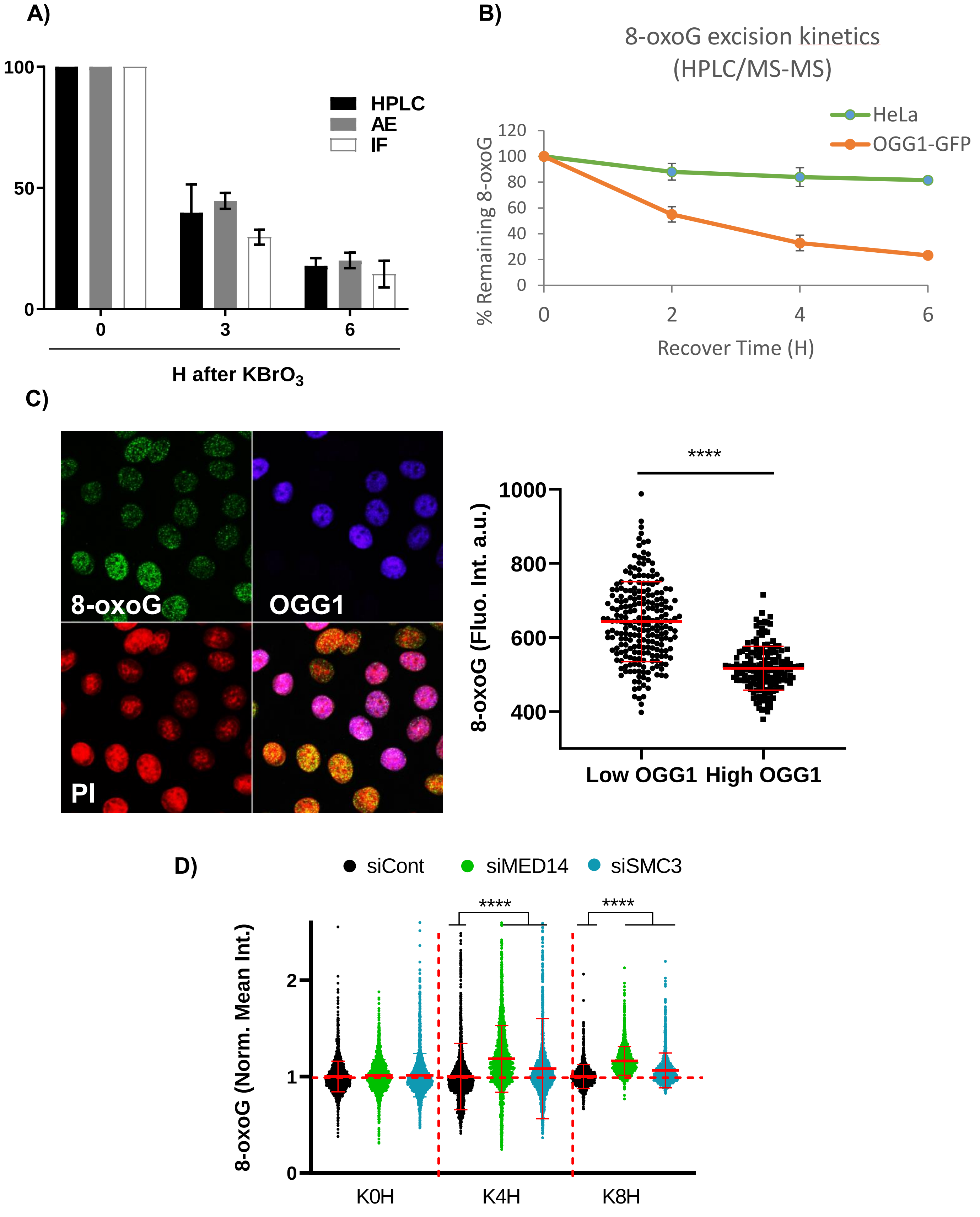

Figure S5: Behavior of Mediator subunits upon exposure to oxidative stress

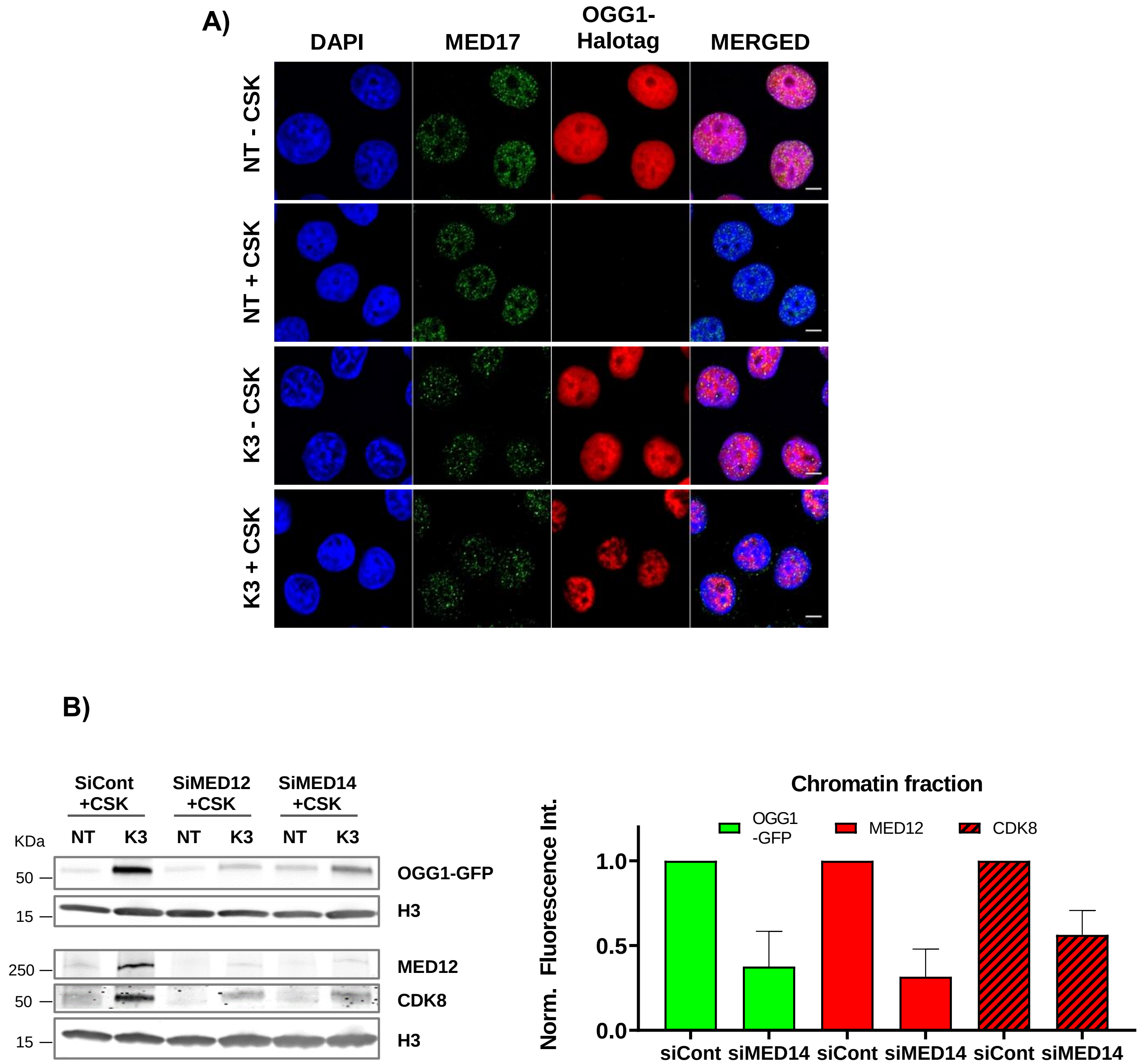

Figure S6. Molecular proximity between OGG1 and the Mediator subunit CDK8 in cells exposed to oxidative stress

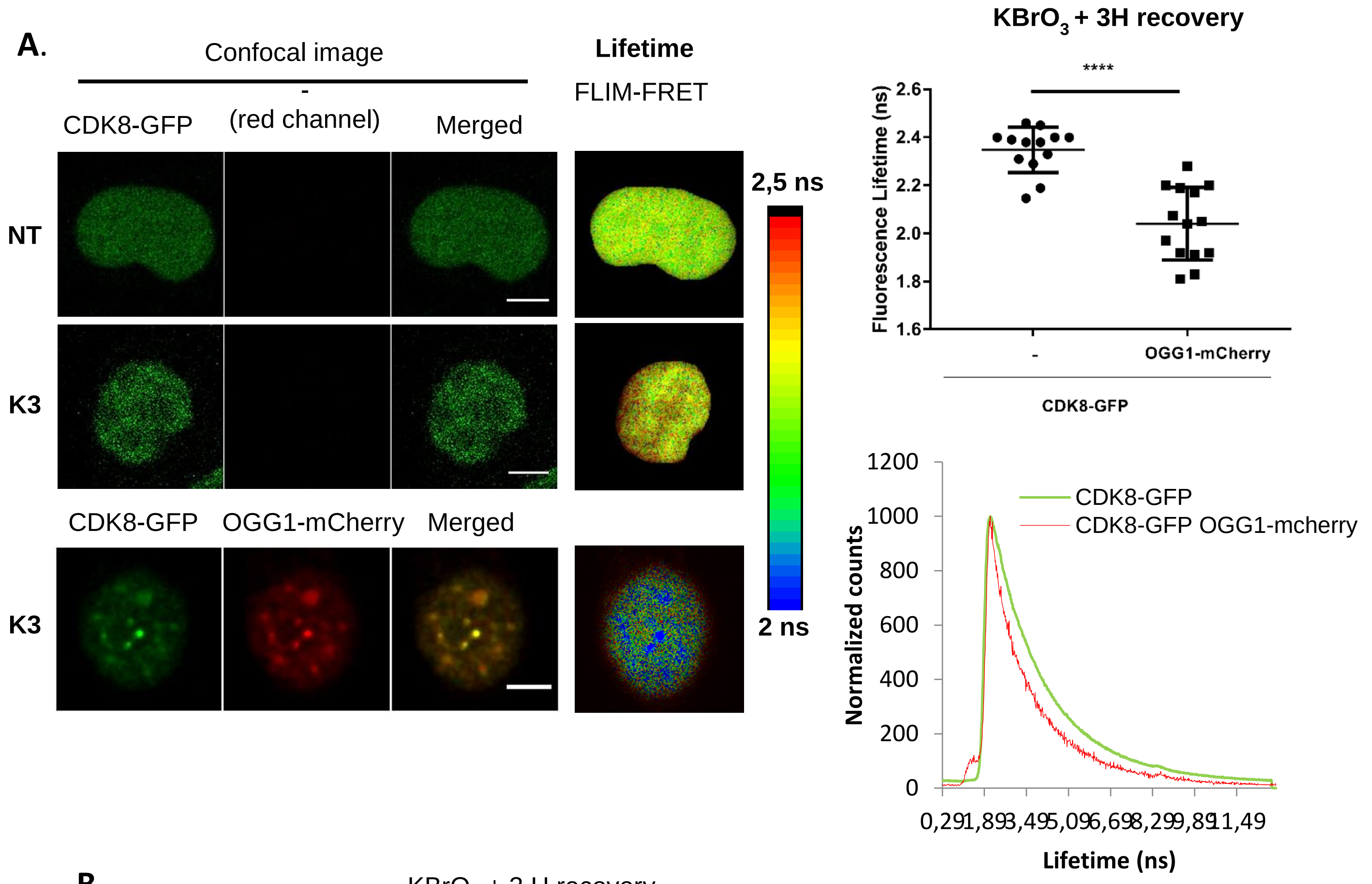

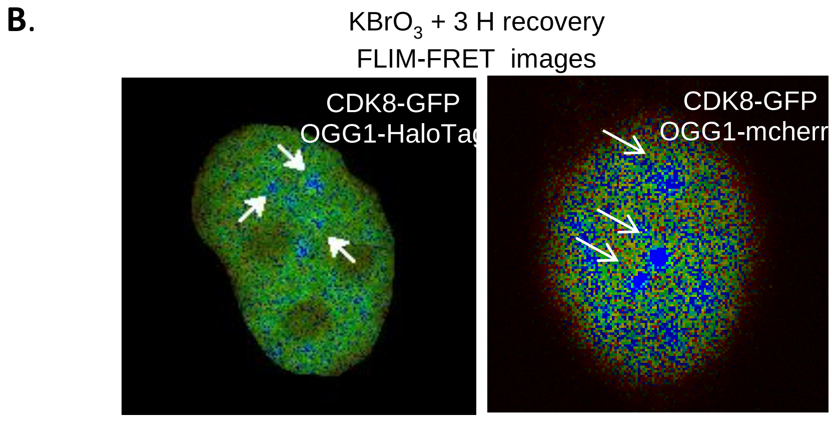

Supplement: gkaa611_Supplemental_Files [file gkaa611_supplemental_files.zip › Lebraud et al NAR SUPPLEMENTARY INFORMATION-revision4-text and FIG.docx.pdf]
